# Macroscopic ordering of helical pores for arraying guest molecules noncentrosymmetrically

**DOI:** 10.1038/ncomms9418

**Published:** 2015-09-29

**Authors:** Chunji Li, Joonil Cho, Kuniyo Yamada, Daisuke Hashizume, Fumito Araoka, Hideo Takezoe, Takuzo Aida, Yasuhiro Ishida

**Affiliations:** 1 Department of Chemistry and Biotechnology, School of Engineering, The University of Tokyo, Hongo, Bunkyo, Tokyo 113-8656, Japan; 2 RIKEN Center for Emergent Matter Science, 2-1 Hirosawa, Wako, Saitama 351-0198, Japan; 3 Department of Organic and Polymeric Materials, Tokyo Institute of Technology, 2-12-1-S8-42 O-okayama, Meguro, Tokyo 152-8552, Japan; 4 PRESTO, Japan Science and Technology Agency, 4-1-8 Honcho, Kawaguchi, Saitama 332-0012, Japan

## Abstract

Helical nanostructures have attracted continuous attention, not only as media for chiral recognition and synthesis, but also as motifs for studying intriguing physical phenomena that never occur in centrosymmetric systems. To improve the quality of signals from these phenomena, which is a key issue for their further exploration, the most straightforward is the macroscopic orientation of helices. Here as a versatile scaffold to rationally construct this hardly accessible structure, we report a polymer framework with helical pores that unidirectionally orient over a large area (∼10 cm^2^). The framework, prepared by crosslinking a supramolecular liquid crystal preorganized in a magnetic field, is chemically robust, functionalized with carboxyl groups and capable of incorporating various basic or cationic guest molecules. When a nonlinear optical chromophore is incorporated in the framework, the resultant complex displays a markedly efficient nonlinear optical output, owing to the coherence of signals ensured by the macroscopically oriented helical structure.

## Abstract (web summary)

Helical nanostructures often exhibit intriguing physical phenomena. Here, the authors fabricate a helical porous material exhibiting both controlled handedness and macroscopic orientation, which promises applications in a wide array of disciplines.

## Graphical abstract

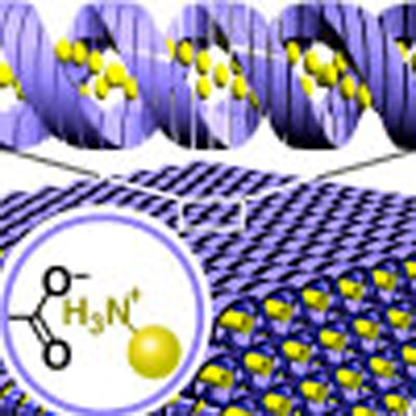

When appropriately functionalized molecules are arranged in one-handed helices, they often display intriguing physical phenomena that never occur in centrosymmetric structures, as represented by second harmonic generation (SHG)^1^,^2^,^3^,^4^ and piezoelectricity^5^,^6^,^7^,^8^. These helices would also give a clue to pursue unexplored predictions, including molecular solenoid effects^9^,^10^. To explore these phenomena further, a key issue is to improve the quality of their output signals. For this purpose, the most straightforward approach is to orient the helices macroscopically so that mutual cancellation of signals is prevented^3^,^4^,^5^,^6^,^8^. Although such hierarchical structures, realizing one-handed helicity and macroscopic orientation at the same time, are rarely obtained, their rational formation would become possible by using a framework with macroscopically oriented helical pores^11^,^12^,^13^,^14^,^15^,^16^,^17^. If this framework serves as a scaffold for arraying various molecules in a macroscopically oriented helical structure by simple host–guest complexation, it should facilitate the exploration of the aforementioned phenomena. However, such a framework has not been developed so far. Even in the case of achiral pores, their macroscopic orientation remains a general challenge^18^,^19^,^20^,^21^,^22^. For the present purpose, individual pores must be helical and capable of precisely positioning the incorporated molecules, which makes this challenge even greater.

With the aim of developing such frameworks, we focused on an advanced version of molecularly imprinted polymers^23^ prepared by the crosslinking of liquid crystals (LCs), which have been pioneered by Gin *et al*.^24^ and are now regarded as a new class of solid-state hosts^25^,^26^,^27^,^28^,^29^,^30^,^31^,^32^. When a multicomponent LC composed of a polymerizable frame unit and a non-polymerizable template unit (for example, Fig. 1, i) is *in situ* crosslinked, the frame units are converted into a polymer framework, while the template units are noncovalently captured in the polymer framework and therefore are exchangeable with other molecules. Owing to the well-controlled structure, thus obtained polymer frameworks exhibit unique functions in conversion^24^, binding^25^,^26^,^27^,^28^,^29^,^30^ and transport^31^,^32^ of guest molecules. As we recently demonstrated, chiral pores are rationally constructed by using chiral template units^27^,^28^. Although previous polymer frameworks were mostly prepared from randomly oriented LCs except for a few examples^31^,^32^, we noted that LCs are dynamic and potentially orientable macroscopically by application of an external stimulus, such as a force or a field^31^,^32^,^33^,^34^,^35^,^36^,^37^,^38^,^39^,^40^. Among these stimuli, the use of a magnetic field has the advantages of being capable of application in a non-destructive and non-contact manner^20^,^32^,^33^,^34^.

Here we report an unprecedented type of polymer framework with macroscopically oriented helical pores, prepared by *in situ* crosslinking of a supramolecular LC preorganized in a magnetic field. This achievement results from our unexpected finding that a chiral liquid crystalline salt we recently developed^27^,^28^ meets all requirements as the precursor of such framework, that is, multicomponent nature, polymerizability^24^,^25^,^26^,^27^,^28^,^29^,^30^,^31^,^32^, orientability^18^,^19^,^20^,^21^,^22^,^31^,^32^,^33^,^34^,^35^,^36^,^37^,^38^,^39^,^40^ and helicity with controlled handedness^11^,^12^,^13^,^14^,^15^,^16^,^17^. Before this work, these features have been achieved separately, but never simultaneously. The resultant polymer framework serves as a versatile scaffold for arraying various molecules in a macroscopically oriented helical structure, thereby offering useful motifs for the exploration of the physical phenomena particular to noncentrosymmetric systems.

## Results

### Synthesis of the macroscopically oriented polymer framework

**Figure 1:**
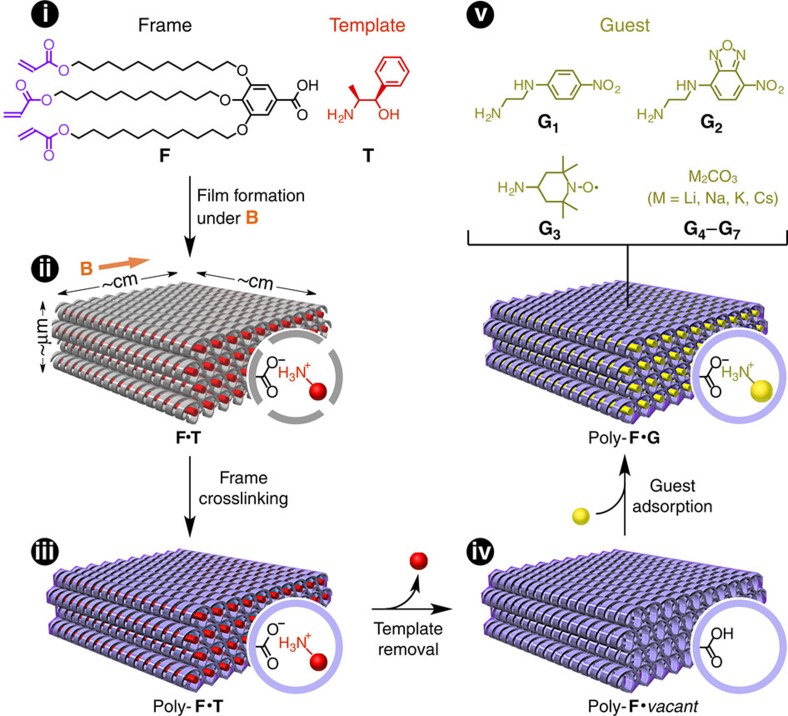
Macroscopically oriented polymer framework with helical pores by *in situ* crosslinking of a magnetically preorganized LC salt. (i) Molecular structure of the frame (polymerizable carboxylic acid, **F**) and template (enantiopure amine, **T**) units that self-assemble into a columnar LC salt. (ii) Processing of the salt (**F·T**) into a macroscopically oriented LC film in a magnetic field. (iii) *In situ* crosslinking of the LC film of **F·T** by radical polymerization to give a polymerized film consisting of poly-**F·T**. (iv) Desorption of **T** from the polymerized film of poly-**F·T** to give a guest-free film of poly-**F·***vacant*. (v) Adsorption of guests **G_1_**–**G_7_** by the guest-free film of poly-**F**·*vacant* to give a guest-exchanged film of poly-**F·G**. **B**, Magnetic field applied during the LC film-preparation process.

**Figure 2:**
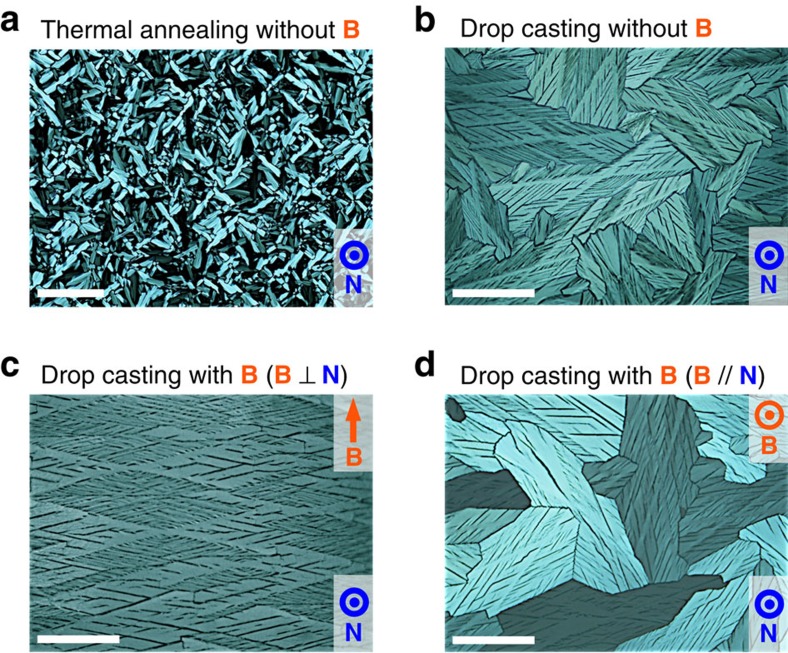
POM images of films of F·T under crossed Nicols. (**a**) LC film (∼10 μm thick) of **F**·**T** processed by slow cooling (–5 °C min^−1^) from an isotropic melt at 130 to 20 °C in the absence of a magnetic field. Scale bar, 100 μm. (**b**–**d**) LC films (∼10 μm thick) of **F**·**T** processed by drop casting of a dichloromethane solution on a glass substrate at 20 °C: in the absence (**b**) and presence of a 10-T magnetic field oriented parallel (**c**) and perpendicular (**d**) to the substrate plane. Scale bar, 500 μm. **B**, Magnetic field applied during the LC film-preparation process. **N**, Normal vector of the film surface.

The polymer framework is prepared with a supramolecular columnar LC material recently reported by our group (Fig. 1, i)^27^,^28^. This consists of a polymerizable carboxylic acid that contains three flexible chains (frame, **F**)^24^ and an enantiopure amine (template, **T**). On mixing in equimolar amounts, these components form a salt (**F**·**T**) that exhibits a stable columnar LC mesophase. In attempt to form a macroscopically oriented structure for the LC salt **F**·**T**, we began with well-established methods, such as thermal annealing and drop casting. When **F**·**T** was slowly cooled from an isotropic molten state at 130 to 20 °C, small, randomly oriented LC domains (∼10 μm) were formed, as confirmed by polarized optical microscopy (POM; Fig. 2a). On the other hand, drop casting of its dichloromethane solution on a glass substrate produced relatively large LC domains, with a size of the order of millimetres and a characteristic crosshatched texture (Fig. 2b). Needless to say, these domains had no orientational regularity at the macroscopic level.

Interestingly, however, the application of a strong magnetic field during the drop-casting process resulted in perfectly controlled orientation of the LC domains over an ∼10-cm^2^-size scale (Fig. 1, ii). For example, **F**·**T** (24.9 mg, 25 μmol) in dichloromethane (500 μl) was cast on a glass substrate (2.5 × 7.5 cm) and slowly concentrated to dryness over 2 h at 20 °C in the presence of a 10-T magnetic field oriented parallel to the substrate plane. As shown in Fig. 2c, the resultant LC film exhibited rhombic patterns of multiple LC domains that aligned along the applied magnetic field to form a continuous two-dimensional (2D) array. Over the entire region of the material, these domains were oriented in the same direction (Supplementary Fig. 1). Because of the high viscosity of LC salt **F**·**T** after perfect drying, the macroscopically oriented film did not undergo structural relaxation, even when the applied magnetic field was turned off. This sluggish relaxation suggests that the present magnetic orientation took place in an intermediate state during the solvent evaporation, where residual solvent lowered the viscosity. In contrast, when a 10-T magnetic field was directed perpendicular to the substrate plane, essentially no effect was brought on the orientation of the LC domains (Fig. 2d). These observations indicate that only the horizontal (in-plane) component of the magnetic field vector contributes to this magnetic orientation, as discussed below.

**Figure 3:**
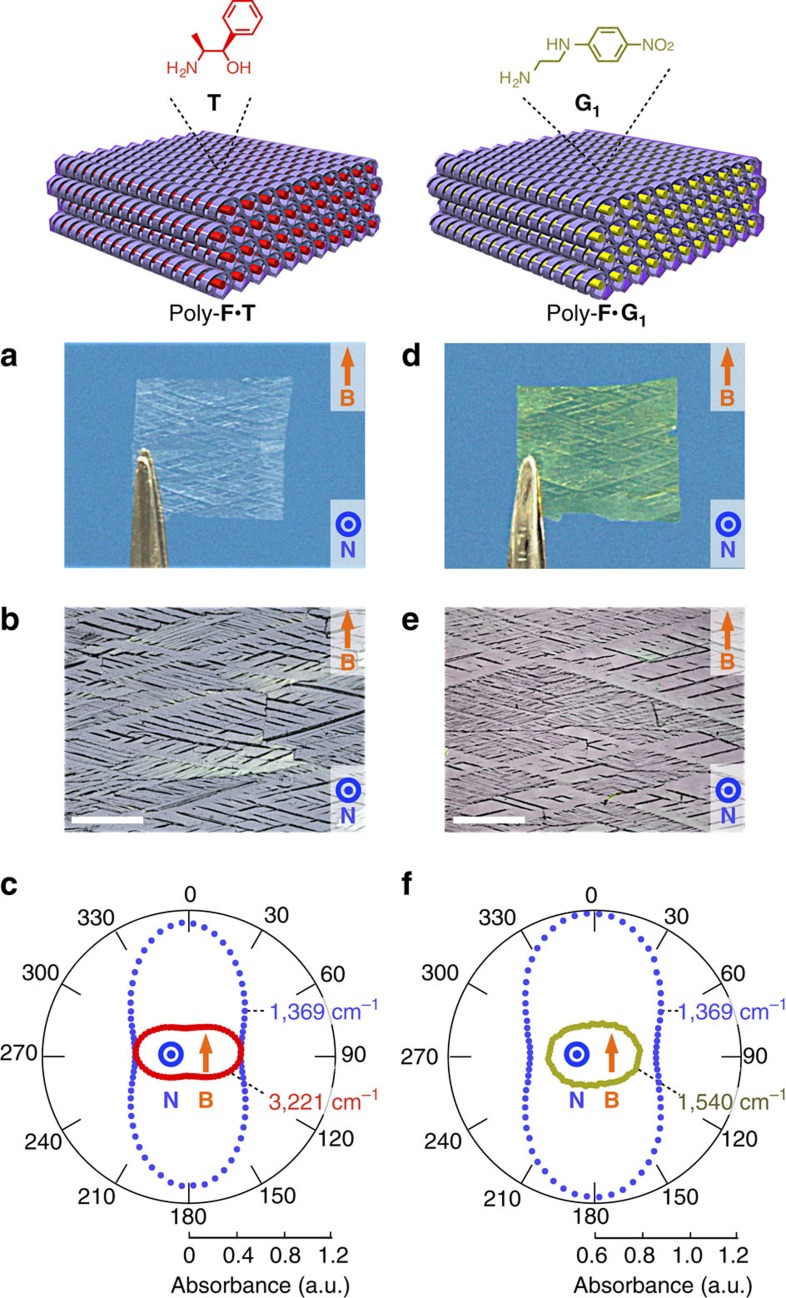
Properties of films with macroscopically oriented structures. (**a**–**c**) Film of poly-**F**·**T** (∼10 μm thick) prepared by *in situ* crosslinking of the LC film of **F**·**T** with a magnetically oriented structure. (**d**–**f**) Film of poly-**F**·**G**_**1**_ (∼10 μm thick) prepared by the guest exchange of the film of poly-**F**·**T** with a magnetically oriented structure. Pictures (**a**,**d**), POM images under crossed Nicols (**b**,**e**) and polar plots of the infrared absorption as a function of the light-polarization angle (**c**,**f**). Scale bar, 500 μm. **B**, Magnetic field applied during the LC film-preparation process. **N**, Normal vector of the film surface.

For covalent fixation of the magnetically oriented structure, the LC film of **F**·**T** was then subjected to *in situ* crosslinking polymerization (Fig. 1, iii). To polymerize the acryloyl groups in **F**, we chose a γ-ray irradiation method^41^ that can be operated without a radical initiator and is a promising method for preserving an organized structure, as we recently disclosed^28^. Otherwise, doping with a radical initiator, even in a small amount (for example, 1 wt% of 2-hydroxy-2-methyl-1-propiophen-1-one), markedly weakened the structural order of the LC film. On irradiating the LC film with γ-ray (6.25 kGy h^−1^) at 20 °C for 16 h, crosslinking polymerization proceeded quantitatively to convert the viscous fluidic **F**·**T** into an insoluble and non-meltable solid (Supplementary Figs 2 and 3) consisting of the salt of polymerized **F** (poly-**F**) and **T** (denoted hereafter as poly-**F**·**T**). This polymer film was flexible and freestanding, so that it was easily peeled off from the glass substrate (Fig. 3a).

### Structural analysis of the framework

Having obtained the macroscopically oriented polymer framework in hand, we investigated its structure at various scales from a macroscopic through the mesoscopic to the molecular. As observed in POM (Fig. 3b) or even by the naked eye (Fig. 3a), the crosshatched texture was extended over the entire region of the polymer film, suggesting that the crosslinking and the peel-off processes had no effect on the macroscopic orientation. When the polymer film was rotated in an in-plane manner, its POM showed a contrast every 45°, giving a dark image when the light-polarization angle with respect to the applied magnetic field was 0° or 90° (Supplementary Fig. 4). The polarized infrared absorption of the film also showed an apparent dependency on the polarization angle, in that the peaks at 1,369 cm^−1^ (–CO_2_^−^) and 3,221 cm^−1^ (–N^+^H_3_) became maximal at the polarization angle of 0° and 90°, respectively (Fig. 3c). From these observations, it is obvious that columnar aggregates of **F** and **T**, which are afforded by salt-pair formation between the –CO_2_H and –NH_2_ groups, lie along the glass surface and are oriented perpendicular or parallel to the applied magnetic field.

**Figure 4:**
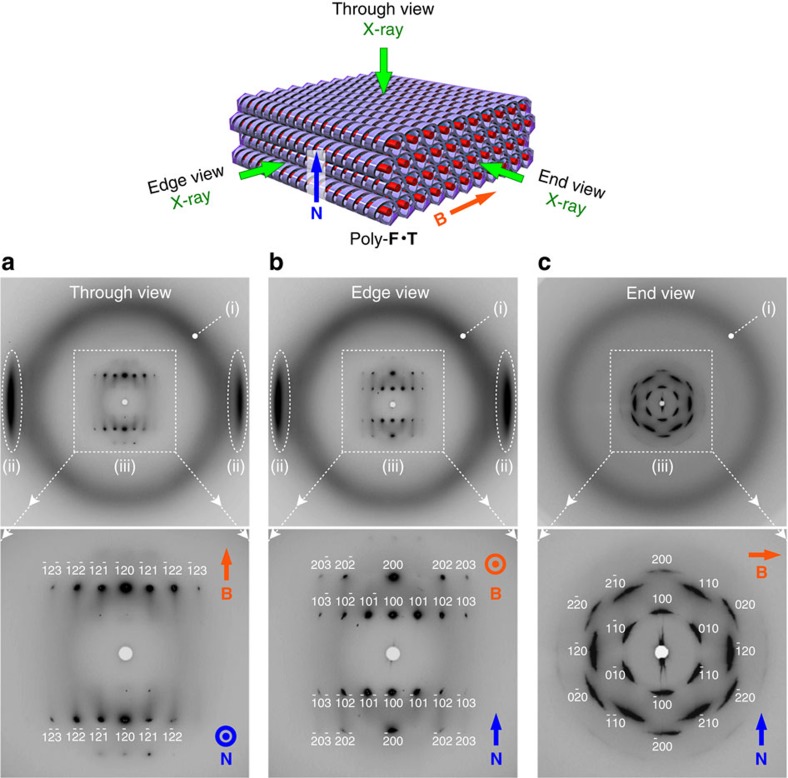
2D X-ray diffraction images of a film of poly-F·T with a macroscopically oriented structure. A square-shaped film (3 × 3 mm, ∼20 μm thick) was used. (**a**) Through view image (X-ray||**N**, X-ray⊥**B**). (**b**) Edge view image (X-ray⊥**N**, X-ray||**B**). (**c**) End view image (X-ray⊥**N**, X-ray⊥**B**). (i), (ii) and (iii) highlight signals due to aliphatic chain packing, π-stacking and 3D lattice, respectively. **B**, Magnetic field applied during the LC film-preparation process. **N**, Normal vector of the film surface.

To examine the structure of the polymer framework in more detail, we performed an X-ray diffraction analysis with a synchrotron radiation source. As shown in Fig. 4 (top), the polymer film was exposed to a beam of X-rays from the directions perpendicular (through view) and in-plane (edge and end views) to the film surface to obtain three-dimensional (3D) structural information^42^. In the in-plane exposure, the X-ray beam was directed parallel (edge view) or perpendicular (end view) to the direction of the magnetic field applied during the preparation of the LC film. In the wide-angle region of the 2D X-ray diffraction images, two types of diffractions characteristic of columnar LCs were observed: a diffuse halo (*d*-spacing, 4.4 Å) due to the loose packing of the aliphatic chains (Fig. 4a–c, i) and a pair of obscure spots (*d*-spacing, 3.6 Å) attributable to the stacking of the *π*-conjugated systems (Fig. 4a,b, ii). Because the diffractions from the *π*-stacking appeared only in the equatorial region of the through and edge views, the *π*-conjugated systems in **F**·**T** are probably stacked in the direction perpendicular to the magnetic field.

To our surprise, a number of sharp spots appeared at regular intervals in the small-angle region (Fig. 4a–c, iii), unlike usual polymer materials. The end view (Fig. 4c) exhibited a pattern of sixfold symmetry, indicating that the columnar objects (diameter, 31.5 Å) are packed in a hexagonal manner (Supplementary Fig. 5c). The patterns in the through (Fig. 4a) and edge views (Fig. 4b) correspond to rectangular lattices, suggesting that the columnar objects have a regular periodicity (distance, 42.6 Å) along the column axis (Supplementary Fig. 5a,b), most likely originating from the helical pitch^43^,^44^. All the reflections could be unambiguously indexed (Fig. 4a–c, iii) by assuming the presence of a 3D hexagonal columnar lattice (*a*=31.5 Å and *c*=42.6 Å). Transmission electron microscopy (TEM) images and the Fourier transform also suggested the hexagonal packing of columnar objects (Supplementary Fig. 7).

The 2D X-ray diffraction images, measured for a relatively large film specimen (3 × 3 mm, ∼20 μm thick) to evaluate its macroscopic structural order, were affected by the angular fluctuation because of the flexible, polydomain nature of the film, as exemplified by the radially broaden spots in Fig. 4c. In addition, the presence of amorphous regions was suggested by a broad scattering at *q*=∼0.04 Å^−1^, where the crystallinity degree of the film was estimated to 82% (Supplementary Fig. 5). Such amorphous regions would be non-negligible particularly at domain boundaries. To remove the effects of polydomain nature, a monodomain clump trimmed from the film was used instead, where much narrower spots were observed (Supplementary Figs 8 and 9). The crystallinity degree was also estimated to be notably higher (92%; Supplementary Fig. 10) but not perfect, probably because of the dynamic nature of the precursor LC and disordering during the *in situ* polymerization.

**Figure 5:**
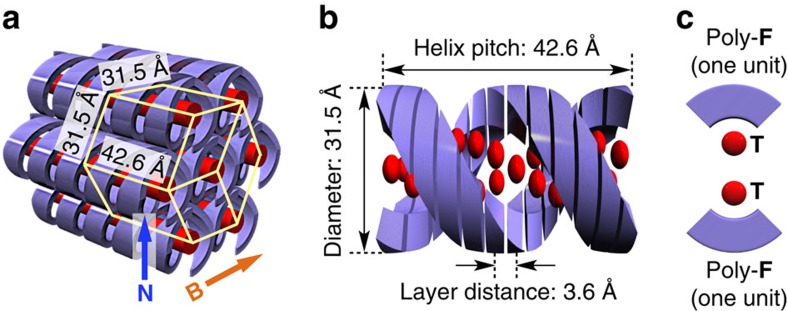
Schematic of the structure of poly-F·T. (**a**) Hexagonally packed helical columns (diameter, 31.5 Å) with structural periodicity along the column axis (distance, 42.6 Å). (**b**) Supramolecular duplex helices formed by the stacking of the repeating unit (shown in **c**). (**c**) Repeating unit consisting of two salt pairs of poly-**F** and **T**. **B**, Magnetic field applied during the LC film-preparation process. **N**, Normal vector of the film surface.

Using the data of the monodomain clump, the space group of poly-**F**·**T** was deduced as follows (Supplementary Methods). After determining the lattice parameters, all indexed reflections were integrated. By calculating the internal residual factor from the reflection intensity and considering symmetry, the Laue group was determined to be 6/*mmm*. Taking account of the systematic extinction rule (Supplementary Fig. 8b), the space group was deduced to be *P*6_1_22 or its enantiomorph *P*6_5_22, which contain right- and left-handed sixfold screw axes, respectively. These space groups also have a twofold screw axis that is perpendicular to the sixfold one, indicating that the whole system is intrinsically apolar and that the sixfold helix forms an antiparallel duplex with another homochiral helix. By assuming that the density of poly-**F**·**T** is ∼1.0 g cm^−3^, we estimated the *Z*-value of the lattice to be 24, indicating the presence of two crystallographically independent pairs of **F**·**T**. In relation to this, the *c* axis lattice parameter (42.6 Å) is exactly 12 times as large as the observed *π*-stacking distance (3.6 Å), indicating that one helical pitch consists of 12 stacking units. Taking account of all the parameters thus obtained, we proposed the structural model shown in Fig. 5, where two salt pairs of **F** and **T** aggregate to form a bow-tie-shaped unit (Fig. 5c). These stack helically with one another (stacking distance, 3.6 Å; rotation angle, 30°) to form a sixfold helix (Fig. 5b). The helical columns lie along the substrate plane and are oriented perpendicular to the applied magnetic field (Fig. 5a).

For further insight into the assembled structure of **F**·**T**, thermal behaviour of an analogous salt **F**·**T′** (**T′**, C2-stereoinverted analogue of **T**) was investigated (Supplementary Fig. 11). As we previously reported, conformational preferences of **T** and **T′** are quite different from each other, where **T** tends to adopt a more flatten conformation (=larger dihedral angle *θ* in Supplementary Fig. 11a) due to the steric hindrance between the phenyl and methyl groups^45^. Such a flatten conformation would facilitate the stacking of the salt pairs of **F** and **T**, as suggested by the diffraction due to *π*-stacking (Fig. 4a,b ii). Indeed, the analogous salt **F**·**T′** cannot form an assembled structure, exhibiting only an isotropic phase (Supplementary Fig. 11b). The X-ray crystal structure of another analogous salt **F′**·**T** (**F′**, analogue of **F** lacking long alkyl chains) is in consistent with the above hypothesis (Supplementary Fig. 12 and Supplementary Data 1). Thus, the salt pairs of **F′** and **T**, adopting a flatten shape, self-assemble via *π*-stacking and hydrogen-bonding interactions. In the crystal structure of **F′**·**T**, the salt pairs assemble into a one-dimensional (1D) array with lateral offsets of the *π*-conjugated systems, while in the LC structure of **F**·**T**, the salt pairs assemble in a helical array, most likely because of the steric hindrance of the long alkyl chains in **F**.

The structural model in Fig. 5 accords with the general tendency of *π*-conjugated systems to orient parallel to an applied magnetic field, and explains the mechanism underlying the present magnetic orientation. Given that an applied magnetic field orients the *π*-conjugated systems in individual molecules of **F** and **T** parallel to the field, it would generate a torque that orients their columnar aggregates perpendicular to the magnetic field. Simultaneously, the columns are induced to lie along the surface of the film, most likely because of the interaction of the columns' aliphatic side chains with the air interface during the drop-casting process. When the magnetic field is applied along the substrate plane, the magnetic and surface effects cooperate to produce a unidirectional orientation^34^. This hypothesis is consistent with the observation that a magnetic field applied perpendicular to the substrate plane had no effect on the structural ordering (Fig. 2d), where both the magnetic and surface effects only defined the columns to orient within the substrate plane and did not restrict their in-plane rotation. We also confirmed that the present magnetic orientation had no effect on the microstructure of the polymer framework; as shown by its 1D SAXS profiles, a film of poly-**F**·**T** with a randomly oriented structure, prepared in the absence of magnet, had the same lattice as that of a magnetically structured film (Supplementary Fig. 6).

### Use of the framework as a solid-state host

By immersing a film of poly-**F**·**T** in acidified ethanol at 20 °C for 10 h, **T** can be quantitatively desorbed from the framework of poly-**F** (Fig. 1, iv), owing to the lack of covalent interactions between **T** and poly-**F** (Supplementary Fig. 13 and Supplementary Table 2). The resultant film, which consisted exclusively of guest-free polymer framework (poly-**F·***vacant*), showed infrared absorption characteristic of a free carboxyl acid (–CO_2_H), while the absorption associated with the carboxylate ion (–CO_2_^−^) disappeared (Supplementary Fig. 15b). Through the desorption of **T,** the macroscopic orientation of the polymer framework was well preserved (Supplementary Fig. 16b). However, the hexagonal columns shrunk in diameter from 31.5 to 30.0 Å (Supplementary Fig. 17c) and lost the periodicity of helical pitch (Supplementary Fig. 17a,b), revealing the flexible nature of the polymer framework^27^.

Because poly-**F·***vacant* contains hollow pores featuring many –CO_2_H groups, the guest-free film readily incorporates various basic or cationic guest molecules **G**_**1**_–**G**_**7**_ (Fig. 1, v). For example, when a film of poly-**F**·*vacant* was immersed at 20 °C for 8 h in a methanolic solution of an amine bearing a *p*-nitroaniline moiety (**G**_**1**_)^46^, the film adsorbed ∼0.9 equivalents of **G**_**1**_ with respect to the –CO_2_H group content of the film (Supplementary Fig. 14 and Supplementary Table 2). The resultant yellow film (Fig. 3d) retained its macroscopic orientation, as confirmed by POM (Fig. 3e). Changes in infrared absorption indicate that this guest binding is driven by salt-pair formation (Supplementary Fig. 15c). Amine **G**_**2**_ bearing a fluorescent moiety and amine **G**_**3**_ bearing a stabilized radical as well as alkali metal ions (**G**_**4**_**–G**_**7**_) were also incorporated as guests in the polymer framework through salt-pair formation with retention of the macroscopic orientation (Supplementary Figs 15 and 16).

As a representative example of a guest-exchanged film, that of poly-**F**·**G**_**1**_ was investigated in more detail. In polarized infrared spectroscopy, its absorption at 1,369 cm^−1^ (–CO_2_^−^) and 1,540 cm^−1^ (–NO_2_) was clearly dependent on the light-polarization angle (Fig. 3f), indicating that the molecular units of **F** and **G**_**1**_ are anisotropically positioned in the macroscopically oriented helical pores. After incorporation of **G**_**1**_, the polymer framework retained its hexagonal columnar packing, and, moreover, recovered the periodicity of helical pitch. In fact, the 2D X-ray diffraction patterns of poly-**F**·**G**_**1**_ (Supplementary Fig. 18) were quite similar to those of poly-**F**·**T** (Fig. 4) for all three views. In addition, lattice parameters of poly-**F**·**G**_**1**_ (column diameter=31.4 Å, helical pitch=42.8 Å) were essentially identical to those of poly-**F**·**T** (column diameter=31.5 Å, helical pitch=42.6 Å), despite the different molecular shapes of **G**_**1**_ and **T**. Such consistency in lattice parameters suggests that a fundamental skeleton of the helical pores might be preserved in poly-**F** even after the helical pitch disordering because of the desorption of **T** (Supplementary Fig. 17). It is probable that molecules of **G**_**1**_ filled the spaces originally occupied by **T** molecules, thereby reducing the structural strain in the polymer framework and recovering the helical pitch^27^. From these observations, we also deduced that the molecules of **G**_**1**_ in the polymer framework were present in a helical arrangement, as in the case of **T**. In this case, a chiral structure emerged from achiral components (**F** and **G**_**1**_) because of the chiral template effect of **T** (ref. 47).

Owing to their push–pull-substituted aromatic systems, *p*-nitroaniline derivatives such as **G**_**1**_ are known to exhibit the second-order nonlinear optical (NLO) properties, when they are arrayed in a noncentrosymmetric (=polar, chiral or both) arrangement^46^,^48^. Because the molecules of **G**_**1**_ confined in poly-**F** are likely to align in helical arrays, as described above, they potentially show second-order NLO properties^1^,^2^,^3^,^4^. Although there have been reported several host systems that induce the NLO output of chromophore guests, most of them contain the contribution of polar structures^46^,^49^. Contrary to them, our polymer framework with a chiral and apolar space group (*P*6_1_22 or *P*6_5_22) would afford NLO output genuinely because of chirality, which is suitable for pursuing nonlinear phenomena in chiral architectures. Furthermore, the macroscopic orientation of poly-**F** would be expected to have positive effects on the NLO output because the NLO signals generated from the ordered structure should be less prone to mutual cancellation and therefore coherent.

**Figure 6:**
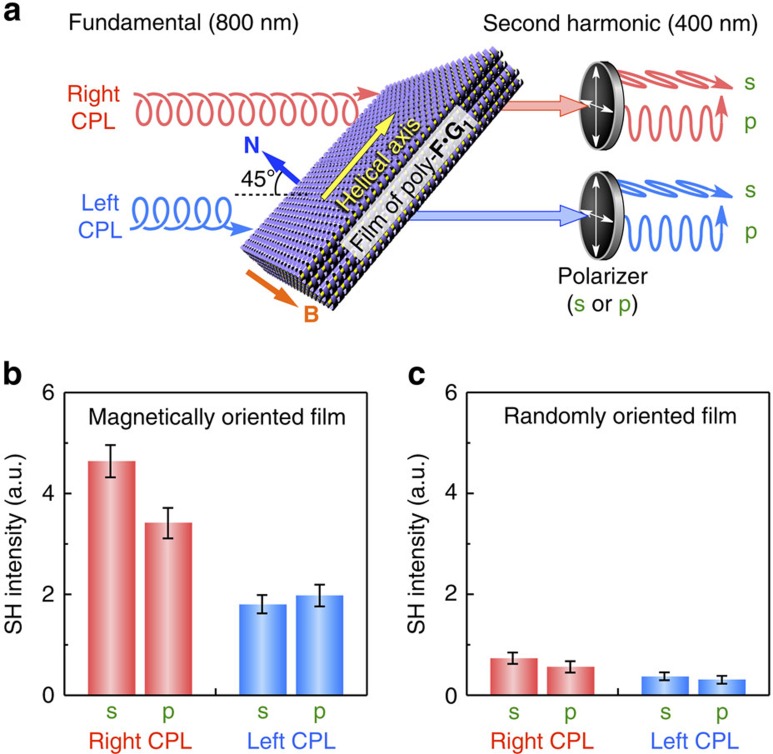
SHG circular dichroism measurement of films of poly-F·G_1_. (**a**) Optical set-up (for details, see Methods and Supplementary Fig. 19). As a fundamental beam, right- (red) or left-handed (blue) CPL (800 nm wavelength) was incident at 45° to a film of poly-**F**·**G**_**1**_ with magnetically oriented structure. The film was set so that the direction of the magnetic field that was applied during the LC film-formation process was perpendicular to the incident plane, that is, the helical axes of the columnar objects were parallel to the incident plane. Generated frequency-doubled light (400 nm wavelength) was detected from the transmitted direction by using an s- or p-analyser. (**b**,**c**) SHG output power for a film of poly-**F**·**G**_**1**_ (∼5 μm thick) with a magnetically oriented structure (**b**) and for an analogous film (∼5 μm thick) with a randomly oriented structure (**c**). Each error bar represents the s.d. of 100 replicate measurements. **B**, Magnetic field applied during the LC film-preparation process. **N**, Normal vector of the film surface.

To confirm this possibility, we measured the SHG of the film of poly-**F**·**G**_**1**_ (Fig. 6a and Supplementary Fig. 19). We used circularly polarized light (CPL) with right- or left-handedness as a fundamental beam, so that we could determine, independently of any effect of birefringence, whether the origin of the SHG was the chiral arrangement induced by the polymer framework or whether it arose from coincidental polar ordering^50^. To detect the SHG circular dichroism clearly, the incident angle was set to 45°, and the s- and p-polarized contents of the SHG output were separately monitored (Methods)^50^. When right-handed CPL (wavelength, 800 nm) was irradiated to a 5-μm-thick film of poly-**F**·**G**_**1**_ with a magnetically oriented structure (Fig. 6a, red), strong SHG output was observed for both of the s- and p-polarized contents (Fig. 6b, red). On switching the handedness of the CPL from right to left (Fig. 6a, blue), the intensity of the SHG reduced by half (Fig. 6b, blue), proving that the SHG observed here is because of the chiral arrangement of **G**_**1**_. As we had conjectured, the macroscopic orientation of the framework had a significant effect on the SHG output. Indeed, the SHG intensity of the film with magnetically oriented structure (Fig. 6b) was seven times greater than that of an analogous 5-μm-thick film with a randomly oriented structure (Fig. 6c).

## Discussion

Although many types of ordered porous materials, such as zeolites, porous silicates and metal–organic frameworks, have been developed so far, our new polymer framework (poly-**F**), prepared by *in situ* crosslinking of a chiral supramolecular LC salt (**F**·**T**) preorganized in a magnetic field (Fig. 1), is the first porous material that realizes helicity with controlled handedness^11^,^12^,^13^,^14^,^15^,^16^,^17^ and macroscopic orientation^18^,^19^,^20^,^21^,^22^,^31^,^32^,^33^,^34^,^35^,^36^,^37^,^38^,^39^,^40^ at the same time. Our framework, capable of incorporating various basic or cationic guest molecules and restricting their positions, is expected to serve as a ‘universal' scaffold for arraying molecules into a helical and macroscopically oriented structure. The resulting arrangement of the guest molecules might be suitable for inducing molecular events to proceed in a directionally controlled and mutually correlated manner, as exemplified by the SHG of a NLO chromophore (**G**_**1**_) demonstrated in this work. Further potential applications of our framework include CPL-emitting devices, piezoelectric materials, chiral magnets, anisotropic ion-conductive materials or membranes for chiral separation.

## Methods

### General

POM was performed on a Nikon model Eclipse LV100POL optical polarizing microscope equipped with a Mettler Toledo model FP90 Central Processor connected with a model FP 82HT hot stage. ^60^Co γ-ray irradiation was carried out in the Takasaki Advanced Radiation Research Institute of Japan Atomic Energy Agency. Film thicknesses were measured using a Mitutoyo model MDQ-30M micrometer. HPLC analysis was performed on a JASCO model PU-980 intelligent HPLC pump equipped with a model UV-970 intelligent ultraviolet–vis detector. Elemental analysis was performed on a Yanaco CHN CORDER MT-6 elemental analyser. ^1^H NMR spectra were measured on a JEOL model NM-Excalibur 500 spectrometer operated at 500 MHz. Infrared spectra were measured on a JASCO model FT/IR-4100 Fourier transform infrared spectrometer with a model ATR PRO450-S-attenuated total reflection equipment. X-ray crystallographic study was carried out using a Rigaku AFC-8 diffractometer with graphite monochromated Mo K*α* radiation at 300 K.

### Materials

3,4,5-Tris(11-acryloyloxyundecyloxy)benzoic acid (**F**)^24^, (1*R*,2*R*)-pseudonorephedrine (**T′**)^51^, 3,5-dimethoxybenzoic acid (**F′**)^52^, *N*-(2-aminoethyl)-*p*-nitroaniline (**G**_**1**_)^46^ and *N*-(7-nitrobenz-2-oxa-1,3-diazol-4-yl)aminoethylamine (**G**_**2**_)^53^ were prepared according to literature methods. Water was obtained from a Millipore model Milli-Q integral water purification system. (1*R*,2*S*)-Norephedrine (**T**) was purchased from TCI and purified by distillation. Other reagents were used as received from Kanto (CH_2_Cl_2_, EtOH, MeOH and HClO_4_ (60% aqueous solution)), Sigma-Aldrich (Cs_2_CO_3_ (**G**_**7**_)), TCI (4-amino-2,2,6,6-tetramethylpiperidine-1-oxyl (**G**_**3**_)) and Wako (HCO_2_H, hexane, Li_2_CO_3_ (**G**_**4**_), K_2_CO_3_ (**G**_**6**_) and Na_2_CO_3_ (**G**_**5**_)).

### Synthesis of a film of poly-F·T with magnetically oriented structure

A solution of salt **F**·**T** (24.9 mg, 25 μmol) in CH_2_Cl_2_ (500 μl) was cast on a glass substrate (2.5 × 7.5 cm). The glass substrate was placed inside a pair of loosely closed Petri dishes that were immediately inserted into the bore of a superconducting magnet with its 10-T field oriented parallel to the substrate plane. The cast solution of **F**·**T** was carefully and slowly concentrated to dryness over 2 h at 20 °C. The resultant material was further dried under reduced pressure (∼1 mm Hg) at 20 °C overnight to remove residual volatiles to give an LC film of **F·T** with a magnetically oriented structure. To achieve *in situ* polymerization of the acryloyl groups in **F**, the LC film on the glass substrate was placed in a glass tube equipped with a three-way cock. The glass tube was evacuated by means of a rotary oil pump (∼1 mm Hg) at 20 °C for 1 h and was purged with argon. The film in the glass tube was then exposed to ^60^Co γ-ray (6.25 kGy h^−1^) at 20 °C for 16 h so that the LC film was converted into a film of poly-**F**·**T** with a magnetically oriented structure. The film was immersed in CH_2_Cl_2_ at 20 °C for a few minutes and then detached from the glass substrate. By using a micrometre gauge, the thickness of the film was estimated to be 10–15 μm. The thickness of the film could be tuned by adjusting the concentration of the **F**·**T** solution and the drop-casting area in the LC film-preparation process. Thicknesses of film samples used for the measurements are summarized in Supplementary Table 1.

### Synthesis of a film of poly-F·T with randomly oriented structure

Salt **F**·**T** was sandwiched between two glass plates and warmed to 130 °C so that the salt turned into an isotropic melt. It was then allowed to cool to 20 °C at 5.0 °C min^−1^ and then left to stand at 20 °C overnight to give an LC film with a randomly oriented structure. For *in situ* polymerization of the acryloyl groups in **F**, the LC film sandwiched between the two glass plates was subjected to ^60^Co *γ*-ray irradiation, as described above, to give a film of poly-**F**·**T** with a randomly oriented structure.

### Desorption of T from a film of poly-F·T

A film of poly-**F·T** (20.1 mg, containing 20.2 μmol of **T** and the –CO_2_H groups) was immersed in a 7.5 M solution of HCO_2_H in EtOH (50 ml) at 20 °C for 10 h. The resultant mixture was separated into the film and the supernatant. The film was then washed with EtOH (5 ml) and dried under reduced pressure (∼1 mm Hg) to give a film of poly-**F**·*vacant*. For the quantification of desorbed **T**, the supernatant and the washings were combined and concentrated to dryness, dissolved in a pH 2.0 aqueous solution of HClO_4_ (4.00 ml) containing L-tyrosine (3.0 mM) as an internal standard, and subjected to HPLC. The concentration of **T** was calculated from the data of authentic solutions of **T** (Supplementary Fig. 13). Column, Daicel CROWNPAK CR (+) (4.6 × 153 mm); eluent, pH 2.0 aqueous HClO_4_; temperature, 20 °C; flow rate, 0.60 ml min^−1^; injection volume, 5.0 μl; detection, UV absorption at 200 nm; elution time, 8.9 (L-tyrosine) and 13.3 min (**T**).

### Adsorption of G_1_–G_7_ by films of poly-F·*vacant*

A film of poly-**F**·*vacant* (10.0 mg, containing 11.8 μmol of the –CO_2_H groups) was immersed in a 2.5 mM solution of **G**_**1**_ in MeOH (5 ml) at 20 °C for 8 h. From the resultant mixture, the film was separated, washed with MeOH (2.5 ml) and dried under reduced pressure (∼1 mm Hg) to afford a film of poly-**F**·**G**_**1**_. For the quantification of the uptake of **G**_**1**_, the film of poly-**F**·**G**_**1**_ was immersed in a 7.5-M solution of HCO_2_H in EtOH (25 ml) at 20 °C for 10 h. From the resultant mixture, the supernatant was separated, while the film was washed with EtOH (2.5 ml). The supernatant and the washings were combined and concentrated to dryness, dissolved in a pH 2.0 aqueous solution of HClO_4_ (2.00 ml) containing L-tyrosine (3.0 mM) as an internal standard and subjected to HPLC analysis. The concentration of **G**_**1**_ was calculated from the data of authentic solutions of **G**_**1**_ (Supplementary Fig. 14). Column, Daicel CROWNPAK CR (+) (4.6 × 153 mm); eluent, pH 2.0 aqueous HClO_4_; temperature, 20 °C; flow rate, 1.00 ml min^−1^; injection volume, 5.0 μl; detection, ultraviolet absorption at 228 nm; elution time, 5.4 (L-tyrosine) and 39.3 min (**G**_**1**_).

The other amino guests (**G**_**2**_ and **G**_**3**_) were adsorbed by similar procedures. Alkali metal ion guests (**G**_**4**_–**G**_**7**_) were adsorbed from a mixture of MeOH and water (90:10, v/v) at 70 °C for 8 h.

### Synchrotron 2D X-ray diffraction measurement at SPring-8 BL45XU

2D X-ray diffraction measurement of the polydomain films and powders of poly-**F**·**T** and poly-**F**·**G**_**1**_ was carried out at BL45XU in SPring-8 (Hyogo, Japan)^54^. Diffraction data were collected using a Rigaku imaging plate area detector model R-AXIS IV++. The incident X-ray beam (1.00 Å wavelength) was monochromated by a diamond (1 1 1) double-crystal monochromator. The sample-to-detector distance was 0.40 m. Scattering vector *q* and position of an incident X-ray beam on the detector were calibrated using several orders of layer reflections from silver behenate (*d*=58.380 Å). Film samples were mounted on nylon fibres (*ϕ*=0.2 mm) and exposed at 20 °C to an X-ray beam for 30 (edge and end views) or 300 s (through view). Powder samples were placed into 1.5-mm-*ϕ* glass capillaries and exposed at 20 °C to an X-ray beam for 50 s.

### Synchrotron 2D X-ray diffraction measurement at SPring-8 BL26B2

2D X-ray diffraction measurement for the determination of the space group of poly-**F**·**T** was carried out at BL26B2 in SPring-8 (ref. 55). Diffraction data were collected using a MarMosaic225 detector. The incident X-ray beam (1.0000 Å wavelength) was monochromated by a Si (1 1 1) double-crystal monochromator. The sample-to-detector distance was 500 mm. A monodomain clump (0.7 × 0.6 × 0.2 mm) trimmed from a film of poly-**F**·**T** was held on a MicroMounts (MiTeGen) that was attached to a brass pin (Supplementary Fig. 8a), mounted on a goniometer head, and exposed to an X-ray beam at 27 °C. A total of 18 frames of data were collected with an oscillation range of 10° and an exposure time of 10 s for each frame (Supplementary Fig. 8b). The total oscillation angle was 180°.

### TEM measurement

TEM measurement was performed on a JEOL model JEM-2100F/SP operated at 200-kV accelerating voltage. Films of poly-**F**·**T** were embedded in EPON812 (TAAB Laboratories Equipment) and sectioned at 20 °C with a diamond knife mounted on a Leica Ultracut UCT ultramicrotome. The resultant sections (∼60 nm thick) were floated on water, retrieved on TEM grids, stained in vapour of a 0.5% aqueous solution of RuO_4_ (TAAB Laboratories Equipment) at 20 °C for 10 min and subjected to TEM observation.

### SHG circular dichroism measurement

SHG circular dichroism measurement^50^ was performed with planar freestanding films of poly-**F**·**G**_**1**_. For simplicity, films thinner than the coherent length (5 μm thick) were used. As the fundamental beam, right- and left-handed CPLs were created by using 800-nm light of a titanium:sapphire laser (averaged power, 200 mW; duration, 200 fs; repetition, 80 MHz). The laser beam was successively passed through a polarizer and a quarter-wave plate, where the rotation angle of the quarter-wave plate with respect to the polarizer was set to be 45° or 225° for the right-handed CPL and 135° or 315° for the left-handed CPL. Thus, created CPL was guided into a sample with 45° incidence. In the case of film with a magnetically oriented structure, the film was set so that the direction of the magnetic field that was applied during the LC film-formation process was perpendicular to the incident plane, that is, the helical axes of the columnar objects were parallel to the incident plane. SHG signals were detected in the transmission direction by a Hamamatsu model H7421-40 cooled photomultiplier tube after successively passing through an infrared-cut filter, an analyser (s or p), and a 400-nm band pass filter. The signals were recorded by a photon-counting system C8855-01 for a window width of 1 s. For optical scheme, see Supplementary Fig. 19.

## 

## Additional information

**Accession codes:** The X-ray crystallographic coordinates for structures reported in this study have been deposited at the Cambridge Crystallographic Data Centre (CCDC), under deposition number 1056043. These data can be obtained free of charge from The Cambridge Crystallographic Data Centre via www.ccdc.cam.ac.uk/data_request/cif.

**How to cite this article:** Li, C. *et al*. Macroscopic ordering of helical pores for arraying guest molecules noncentrosymmetrically. *Nat. Commun.* 6:8418 doi: 10.1038/ncomms9418 (2015).

## Supplementary Material

Supplementary Information Supplementary Figures 1-19, Supplementary Table 1-2, Supplementary Methods and Supplementary References

Supplementary Data 1 Supplementary Data 1
