# Correction: Corrigendum: Macroscopic ordering of helical pores for arraying guest molecules noncentrosymmetrically

**DOI:** 10.1038/ncomms9811

**Published:** 2015-10-26

**Authors:** Chunji Li, Joonil Cho, Kuniyo Yamada, Daisuke Hashizume, Fumito Araoka, Hideo Takezoe, Takuzo Aida, Yasuhiro Ishida

10.1038/ncomms9418

The Cambridge Crystallographic Data Centre (CCDC) accession code provided in this article is incorrect; the correct deposition number is 1059112.

