# Supplementary material for: Macroscopic ordering of helical pores for arraying guest molecules noncentrosymmetrically
Source: Nat Commun. 2015 Sep 29;6:8418. doi: 10.1038/ncomms9418 (PMC4598726; doi:10.1038/ncomms9418)
Supplement: Supplementary Information — Supplementary Figures 1-19, Supplementary Table 1-2, Supplementary Methods and Supplementary References [file ncomms9418-s1.pdf]

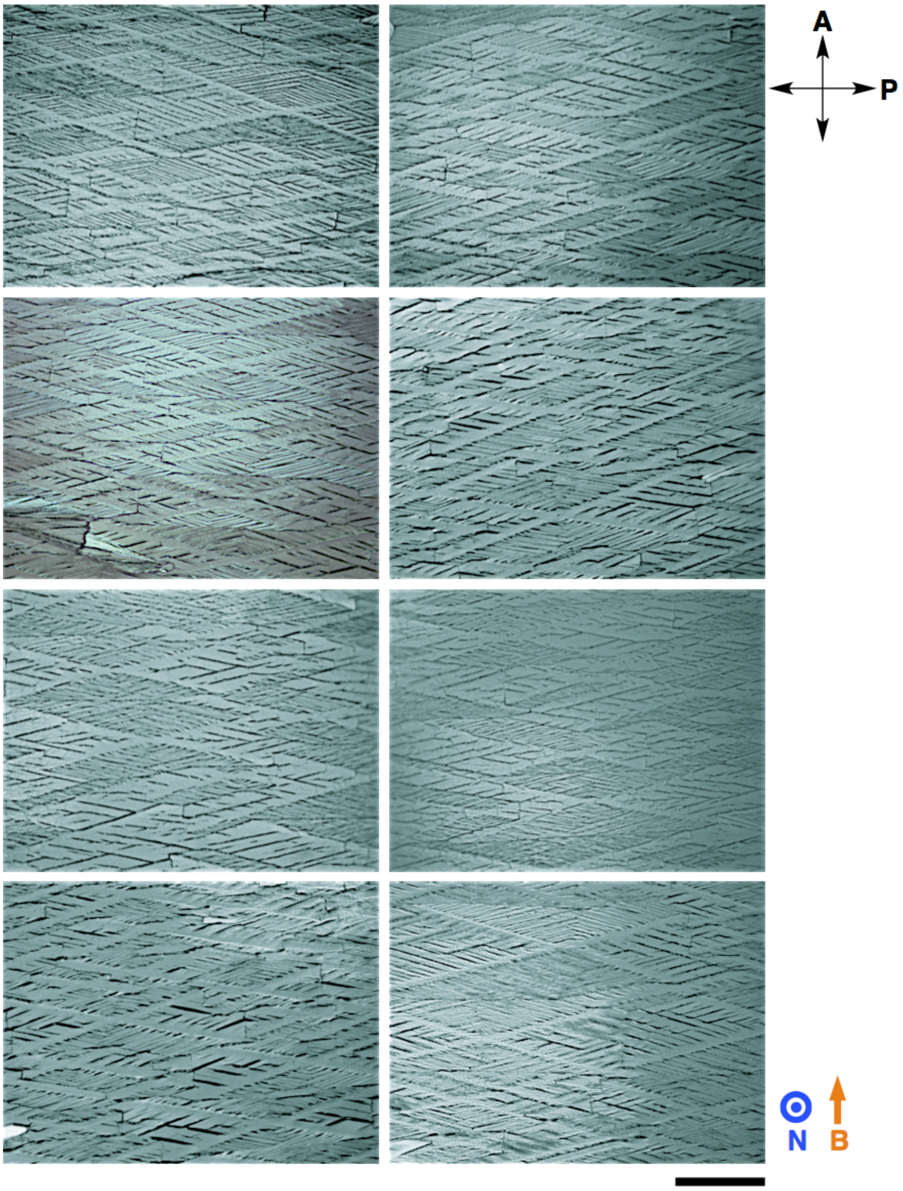

Supplementary Fig. 1 | POM images (under crossed Nicols) of a film of the liquid crystalline salt **F•T** with a magnetically oriented structure (~10 μm thick) at arbitrarily selected viewing fields. **B**: Magnetic field applied during the LC film-preparation process. Scale bar: 500 μm. **N**: Normal vector of the film surface. **P**: Polarizer direction. **A**: Analyzer direction.

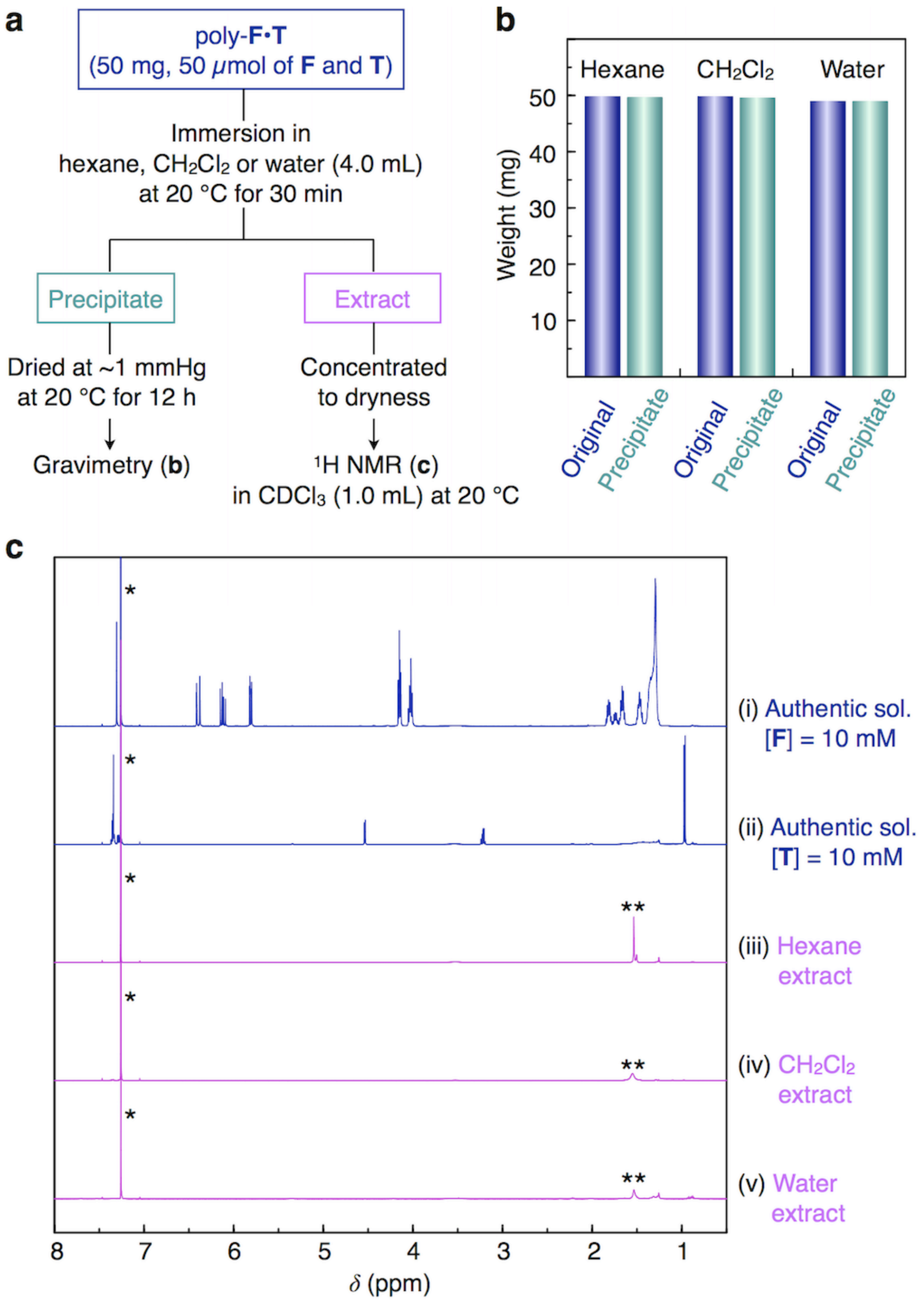

Supplementary Fig. 2 I Solubility tests of a film of poly-**F•T** with a magnetically oriented structure. **a**, Procedure of the solubility tests. **b**, Changes in the weight of the films of poly-**F•T** through the immersion in hexane, CH_2Cl_2 and water. **c**, ^{1}H NMR spectra in $CDCl_3$ at 20 °C of the authentic solutions of **F** (i) and **T** (ii) and the extracts of the immersion in hexane (iii), CH_2Cl_2 (iv) and water (v). In **c**, * and ** denote the signals of residual $CHCl_3$ and H_2O, respectively.

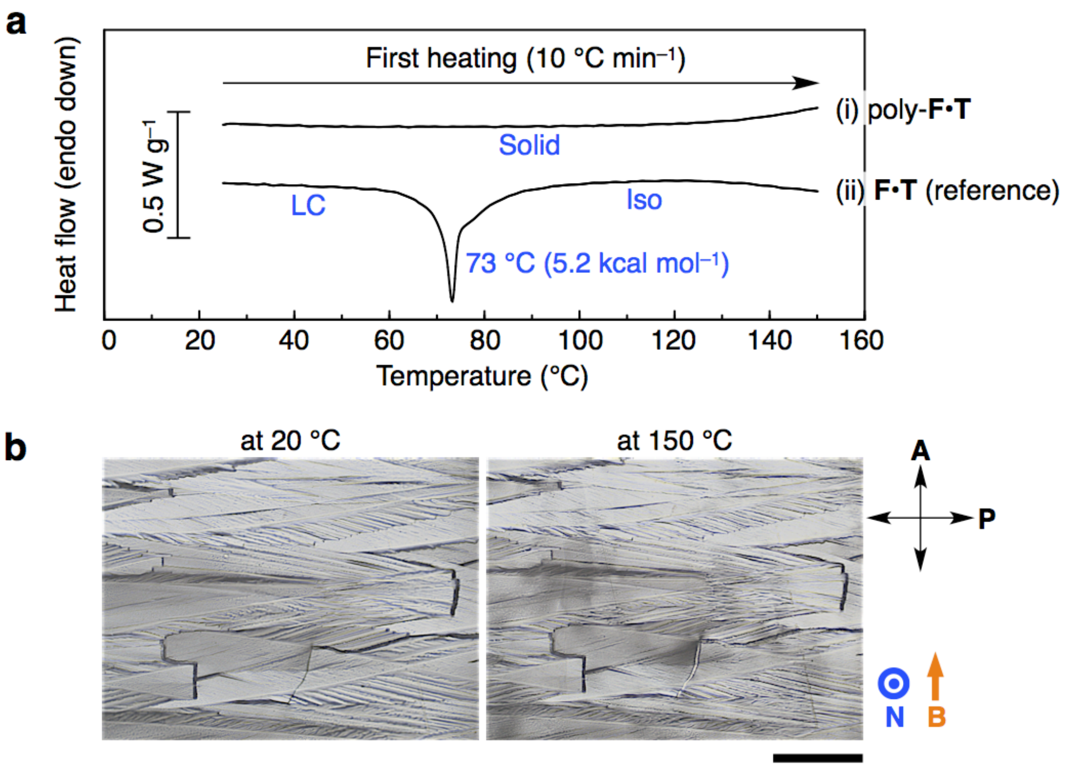

Supplementary Fig. 3 | Meltability tests of a film of poly-**F•T** with a magnetically oriented structure (~10 μm thick). **a**, DSC traces at the first heating (scanning rate, 10 °C min^{-1}) of poly-**F•T** (i) and unpolymerized **F•T** as a reference (ii). Phases, transition temperature and enthalpy change (in parentheses) are indicated in blue. **b**, POM images of poly-**F•T** at the first heating (scanning rate, 10 °C min^{-1}) taken at 20 and 150 °C. Scale bar: 500 μm. **B**: Magnetic field applied during the LC film-preparation process. **N**: Normal vector of the film surface. **P**: Polarizer direction. **A**: Analyzer direction.

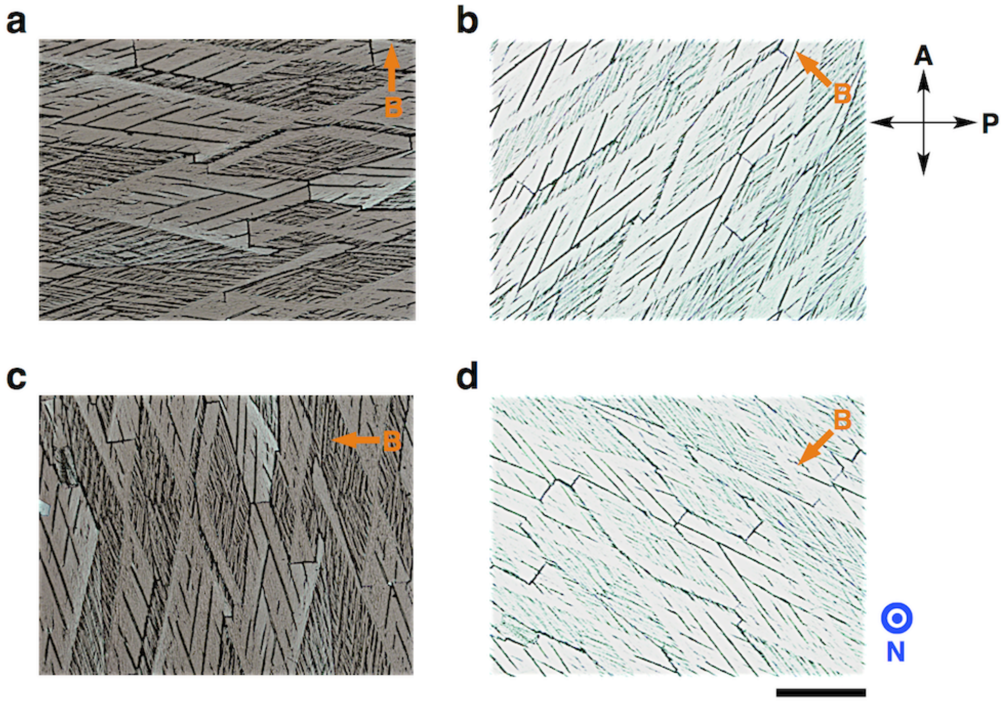

Supplementary Fig. 4 | POM images (under crossed Nicols) of a film of poly-**F•T** with a magnetically oriented structure (~10 μm thick) with varying the angle between **A** and **B**. **a**, 0°. **b**, 45°. **c**, 90°. **d** 135°. Scheme Bar: 500 μm. **B**: Magnetic field applied during the LC film-preparation process. **N**: Normal vector of the film surface. **P**: Polarizer direction. **A**: Analyzer direction.

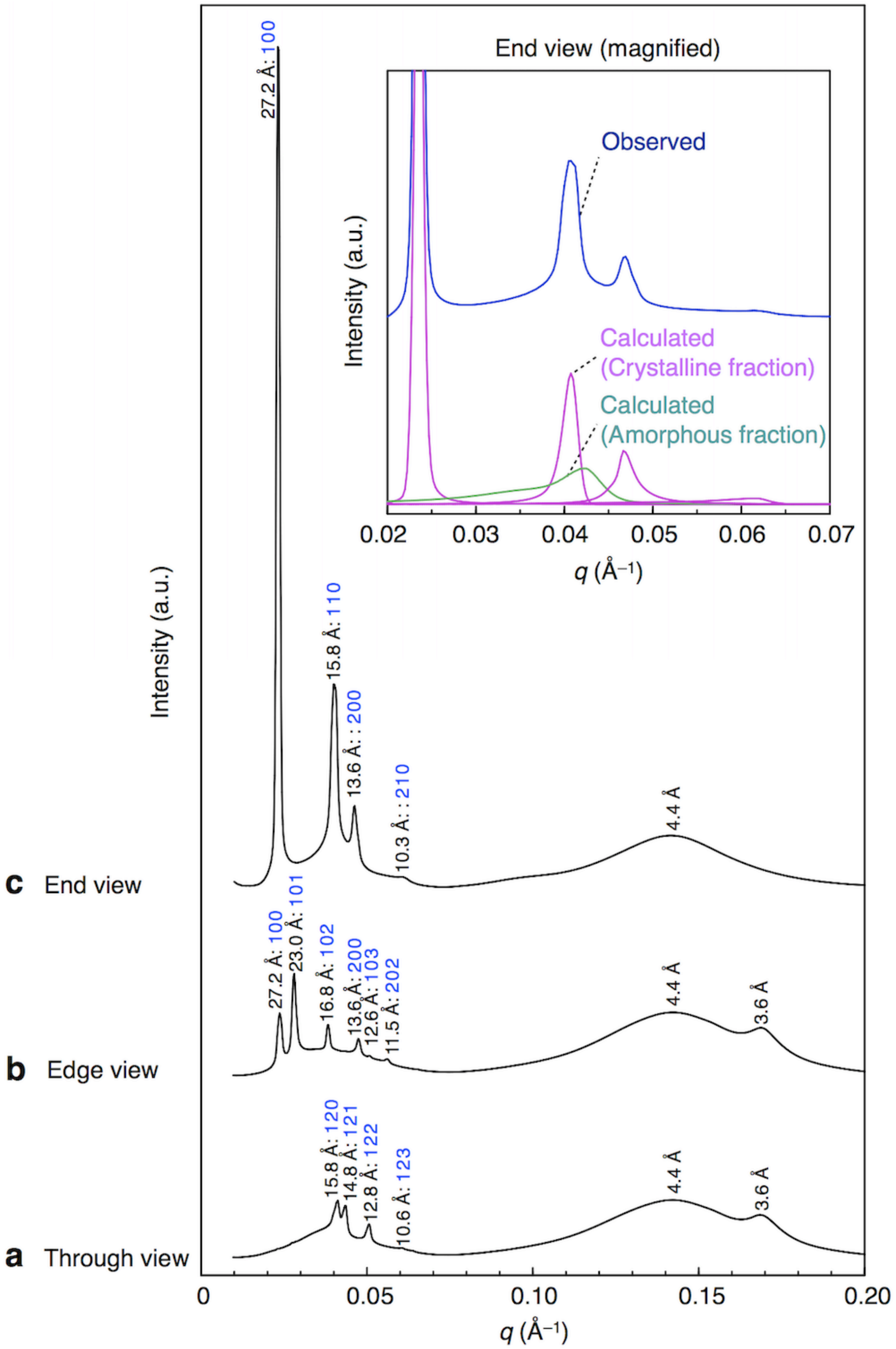

Supplementary Fig. 5 | 1D XRD profiles of a film of poly-**F•T** with a magnetically oriented structure (3 mm × 3 mm, ~20 μm thick). **a**, Through view (X-ray // **N**, X-ray ⊥ **B**). **b**, Edge view (X-ray ⊥ **N**, X-ray // **B**). **c**, End view (X-ray ⊥ **N**, X-ray ⊥ **B**). Miller indices are given in blue. Inset: a small-angle region (q = 0.02–0.07 Å^{-1}) of the 1D XRD profile of the end view (**c**), where the observed data (navy) were curve-fitted and decomposed into four crystalline (magenta) and an amorphous (green) peaks using Rigaku PDXL software[S1].

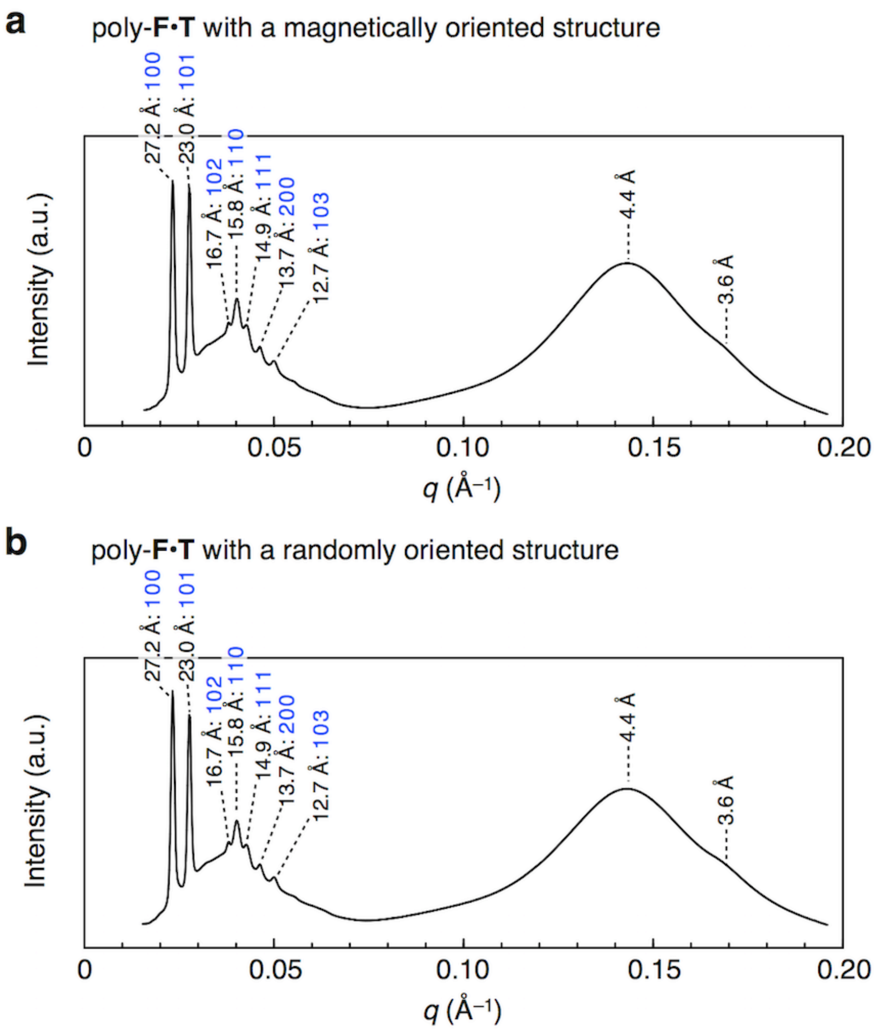

Supplementary Fig. 6 | 1D XRD profiles of powdered samples of poly-**F•T**. **a**, Powder prepared from a film of poly-**F•T** with a magnetically oriented structure. **b**, Powder prepared from a film of poly-**F•T** with a randomly oriented structure. Miller indices are given in blue.

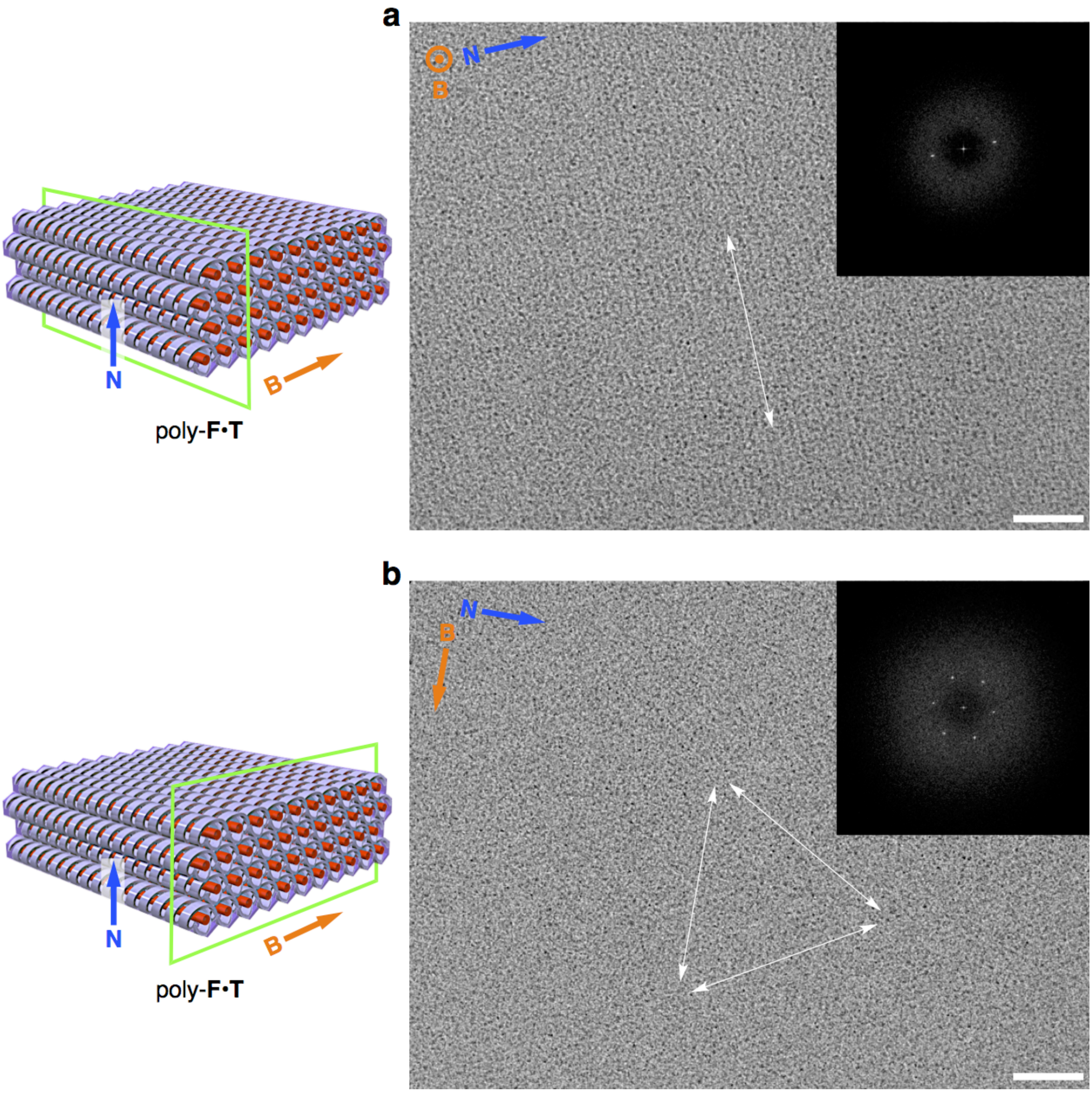

Supplementary Fig. 7 | TEM images of microtomed sections of poly-**F•T** with a magnetically oriented structure. **a**, Microtomed perpendicular to **B**. **b**, Microtomed parallel to **B**. **B**: Magnetic field applied during the LC film-preparation process. **N**: Normal vector of the film surface. Scale bar: 20 nm. White arrow: guide for the eye to show the direction of periodic lines. Inset: Fourier transform of the image.

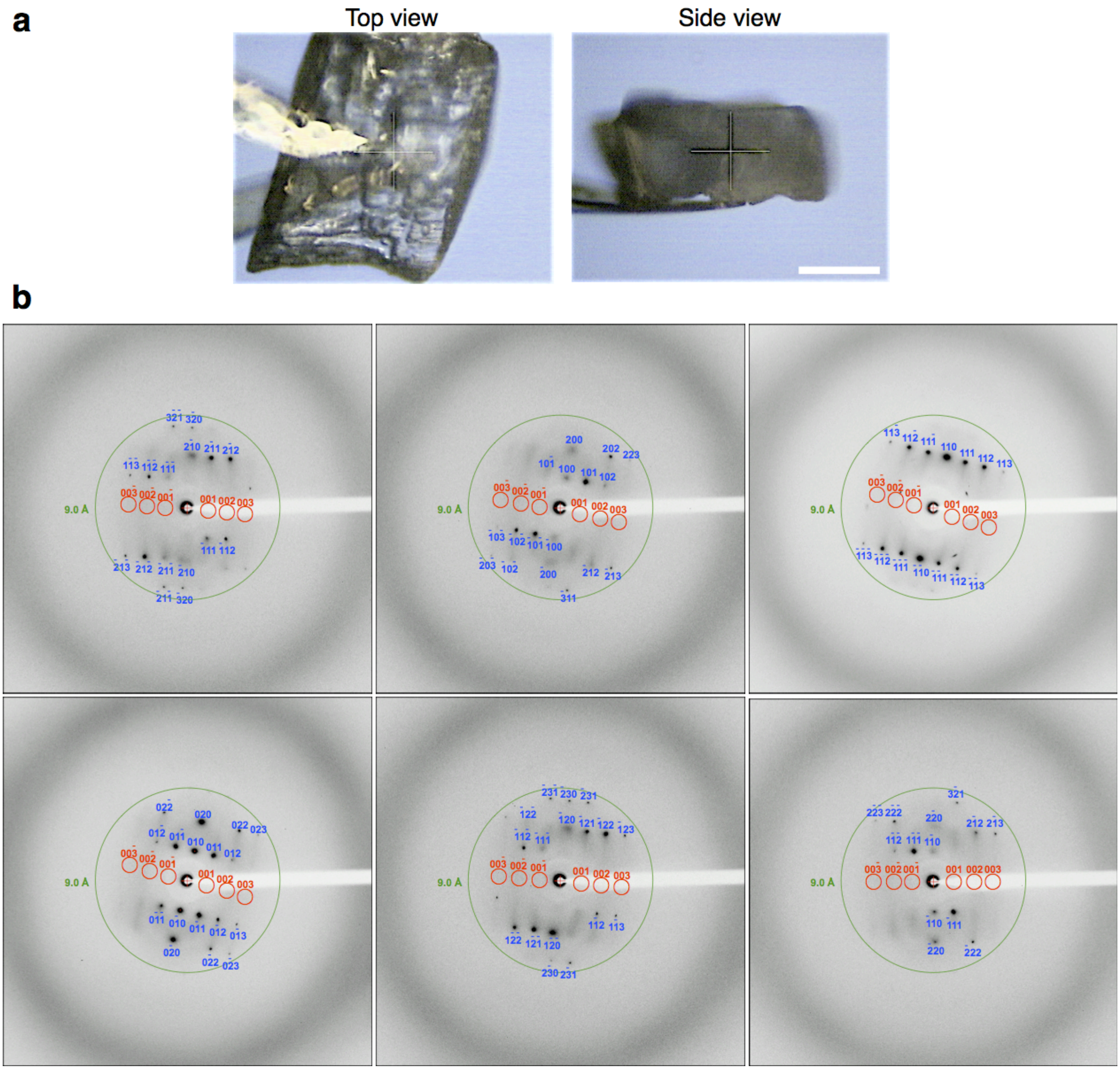

Supplementary Fig. 8 | 2D XRD measurement for a monodomain clump of poly-**F•T** (0.7 mm × 0.6 mm × 0.2 mm) for the determination of the space group of poly-**F•T**. **a**, Pictures of a monodomain clump trimmed from a film of poly-**F•T** with a magnetically oriented structure. Scale bar: 0.2 mm. **b**, Typical frames of the collected data. The red circles display the predicted positions for the 0 0 1, 0 0 2 and 0 0 3 reflections. The green circle indicates 9.00 Å resolution ($2\theta = 6.0°$). Miller indices are given in blue.

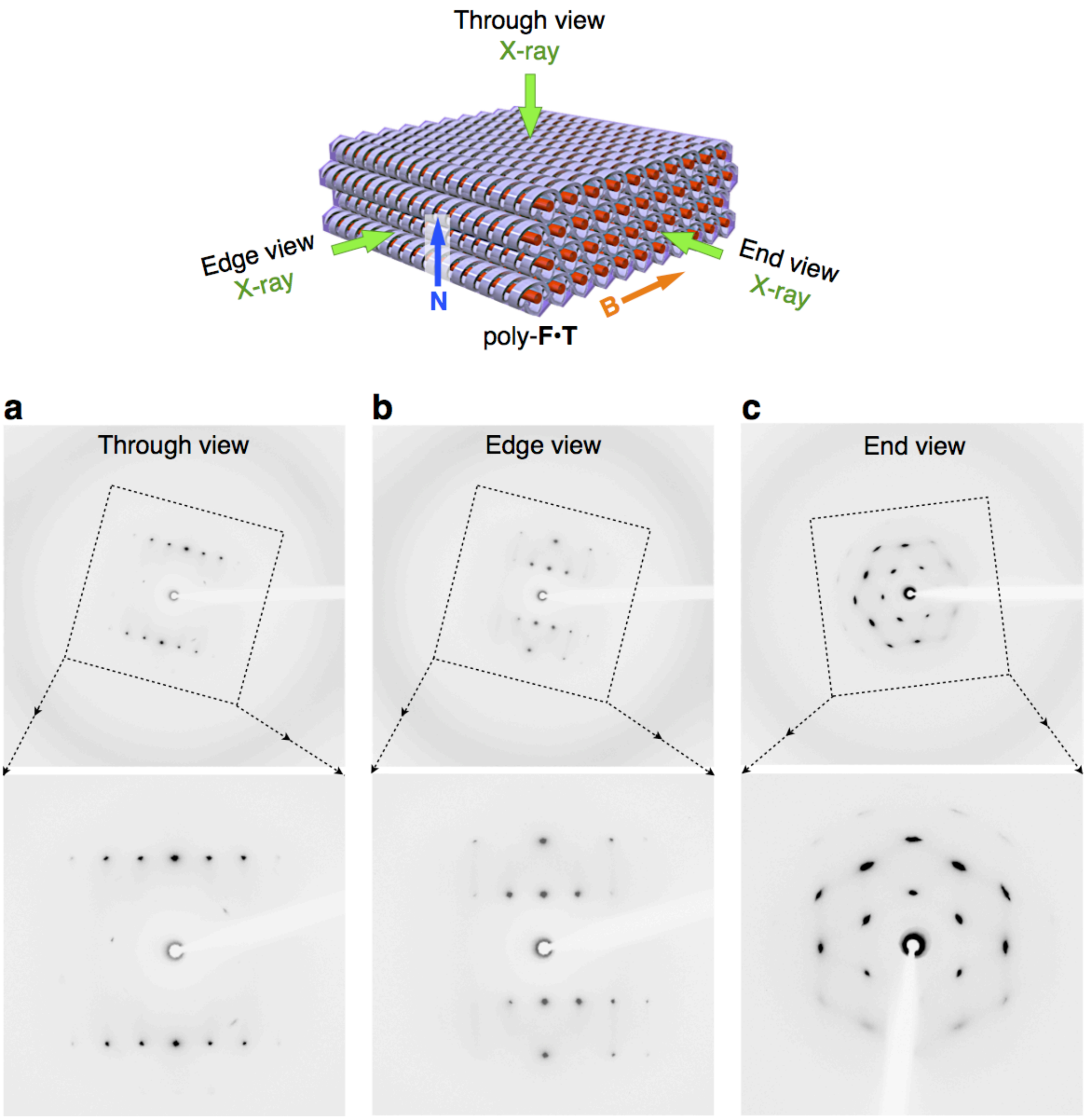

Supplementary Fig. 9 | 2D XRD images of a monodomain clump of poly-**F•T** (0.7 mm × 0.6 mm × 0.2 mm). **a**, Through view image (X-ray // **N**, X-ray ⊥ **B**). **b**, Edge view image (X-ray ⊥ **N**, X-ray // **B**). **c**, End view image (X-ray ⊥ **N**, X-ray ⊥ **B**). **B**: Magnetic field applied during the LC film-preparation process. **N**: Normal vector of the film surface.

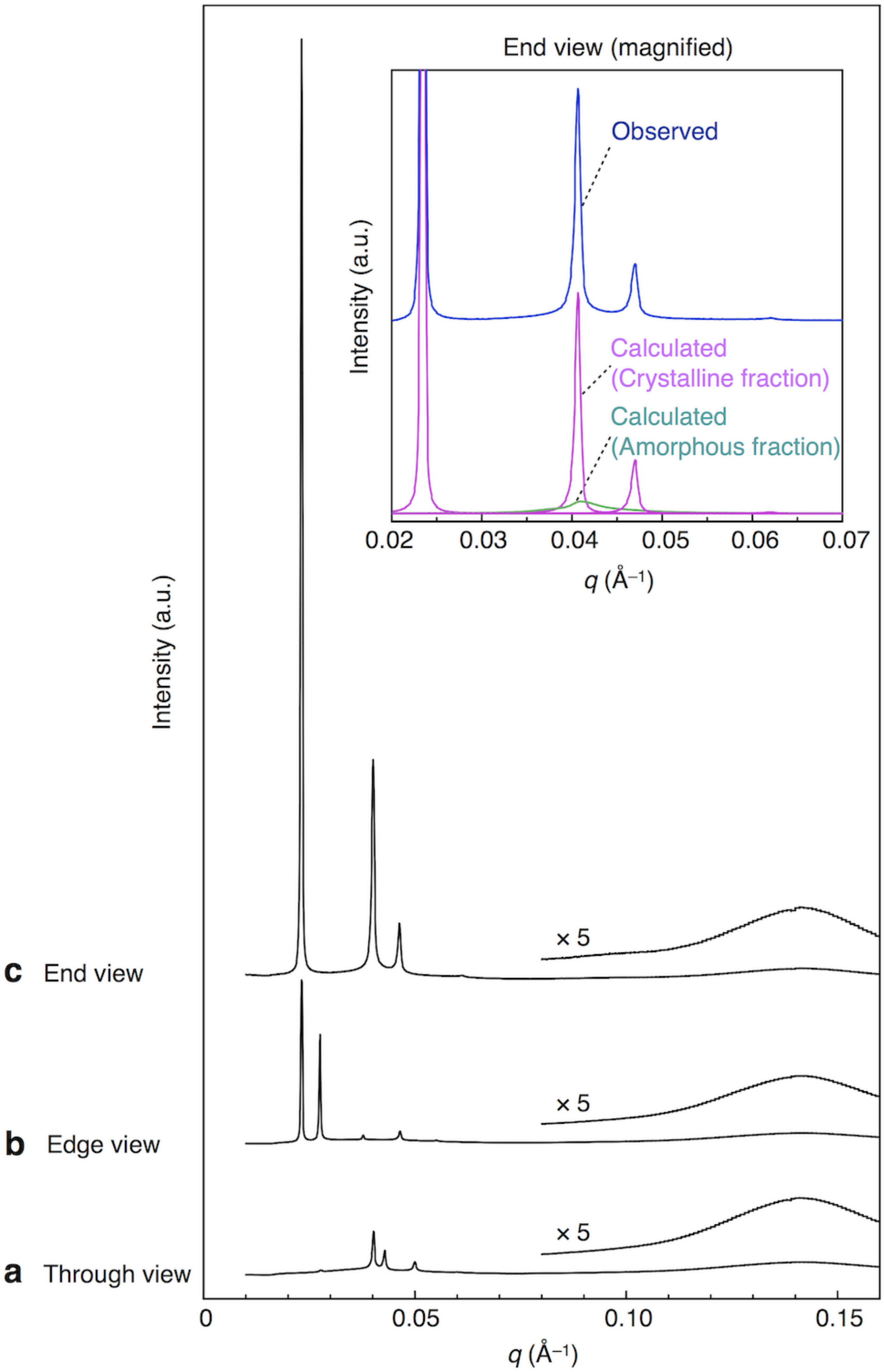

Supplementary Fig. 10 l 1D XRD profiles of a monodomain clump of poly-**F•T** (0.7 mm × 0.6 mm × 0.2 mm). **a**, Through view (X-ray // **N**, X-ray ⊥ **B**). **b**, Edge view (X-ray ⊥ **N**, X-ray // **B**). **c**, End view (X-ray ⊥ **N**, X-ray ⊥ **B**). For Miller indices, see Supplementary Fig. 5. Inset: a small-angle region (q = 0.02–0.07 $Å^{-1}$) of the 1D XRD profile of the end view (**c**), where the observed data (navy) were curve-fitted and decomposed into four crystalline (magenta) and one amorphous (green) peaks using Rigaku PDXL software[S1].

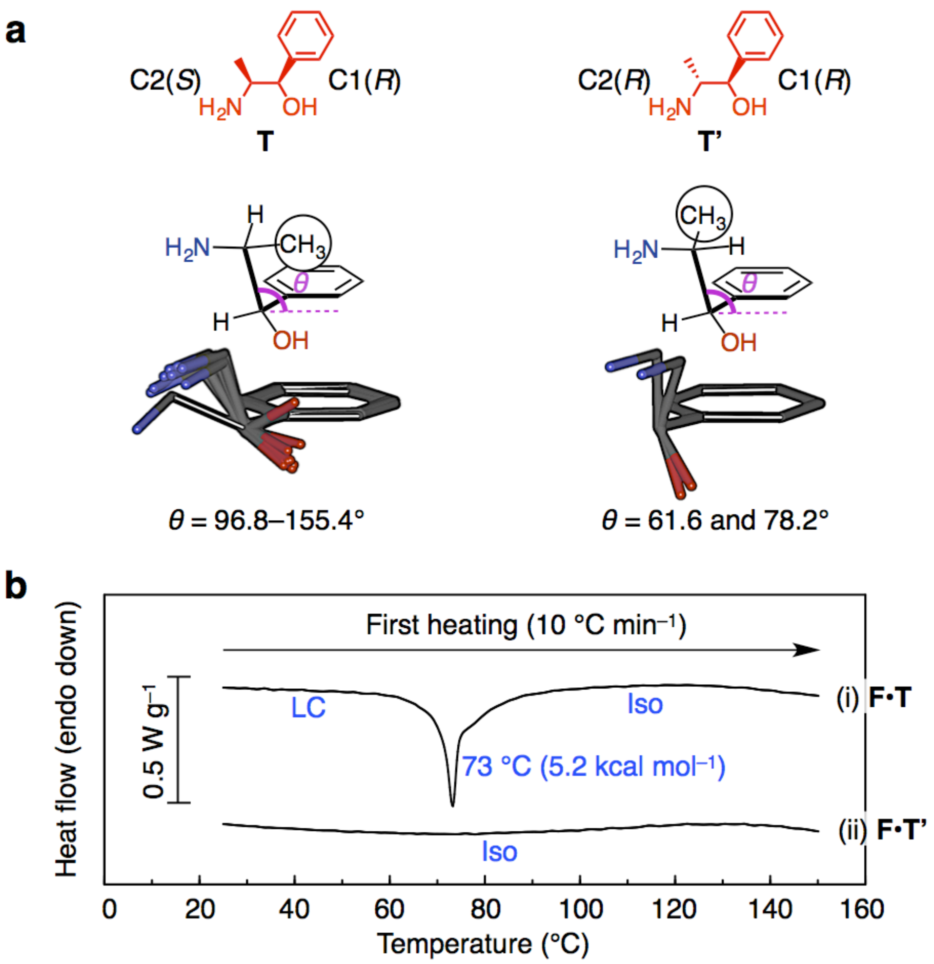

Supplementary Fig. 11 | Thermal behaviors of the LC salt **F•T** and an analogous salt **F•T’**. **a**, Conformational preferences of **T** and **T’**. Superimposition of the structures retrieved from the Cambridge Structure Database (CSD) for the salts of **T** (CSD entry code: *amagaw*, *jastus*, *mixpeac*, *nephcl*, *qawsuc*, *ugeyua*, *vejqeg*) and the salts of **T’** (CSD entry code: *gevneb*, *jasvaa*)[S2]. Hydrogen atoms and the C2-methyl groups are omitted for clarity. **b**, DSC traces at the first heating (scanning rate, 10 °C min^{-1}) of **F•T** (i) and **F•T’** (ii). Phases, transition temperature and enthalpy change (in parentheses) are indicated in blue.

a

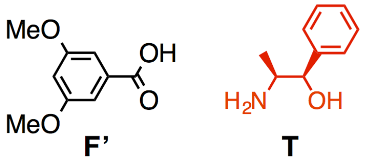

Formula (weight)	$C_{18}H_{23}NO_5$ (333.38)
Lattice	Primitive monoclinic<br>*a* = 12.6748(3) Å<br>*b* = 5.30550(10) Å<br>*c* = 13.6447(3) Å<br>*β* = 106.4792(8)°<br>*V* = 879.86(3) $Å^3$
Space group	$P2_1$ (#4)
Z value	2
Final *R* indices [$I > 2\sigma(I)$]	R_1 = 0.0496, wR_2 = 0.1364
R indices (all data)	R_1 = 0.0642, wR_2 = 0.1484

b

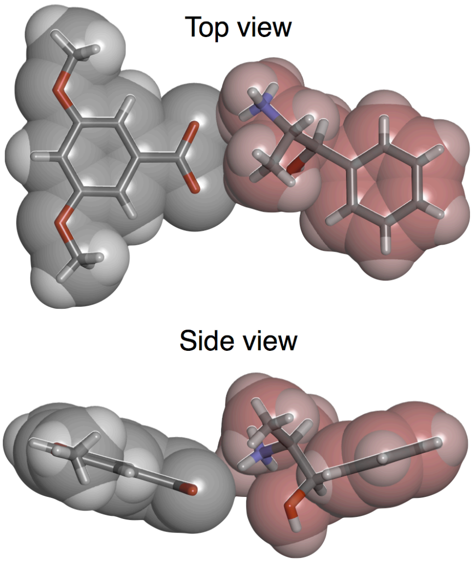

c

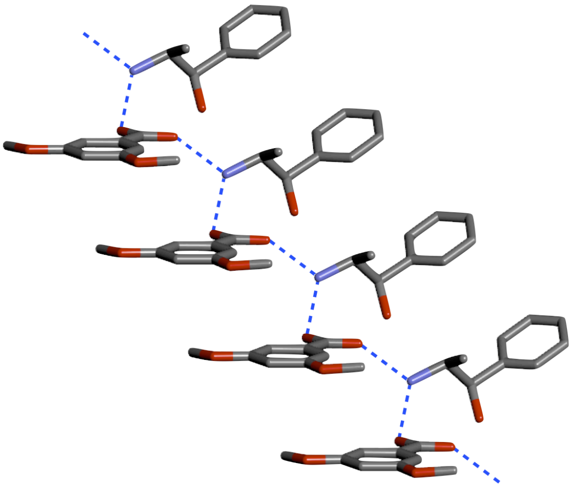

d

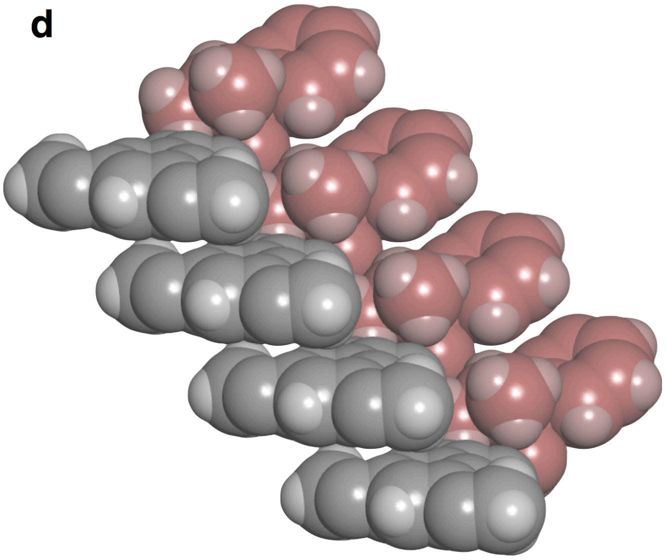

Supplementary Fig. 12 | X-ray crystal structure of an analogous salt **F’•T** (CCDC deposition no. 1059112). A single crystal of **F’•T** used for the X-ray crystallographic study was obtained by vapor diffusion from tetrahydrofran and hexane. **a**, Chemical structures of the salt **F’•T** and summary of the crystal structure. **b**, A salt-pair unit of **F’** and **T**. **c**,**d**, Assembly of the salt pairs of **F’** and **T** in stick (**c**) and CPK (**d**) representations. In **c**, hydrogen bonds are indicated by blue dotted lines, and hydrogen atoms are omitted for clarity.

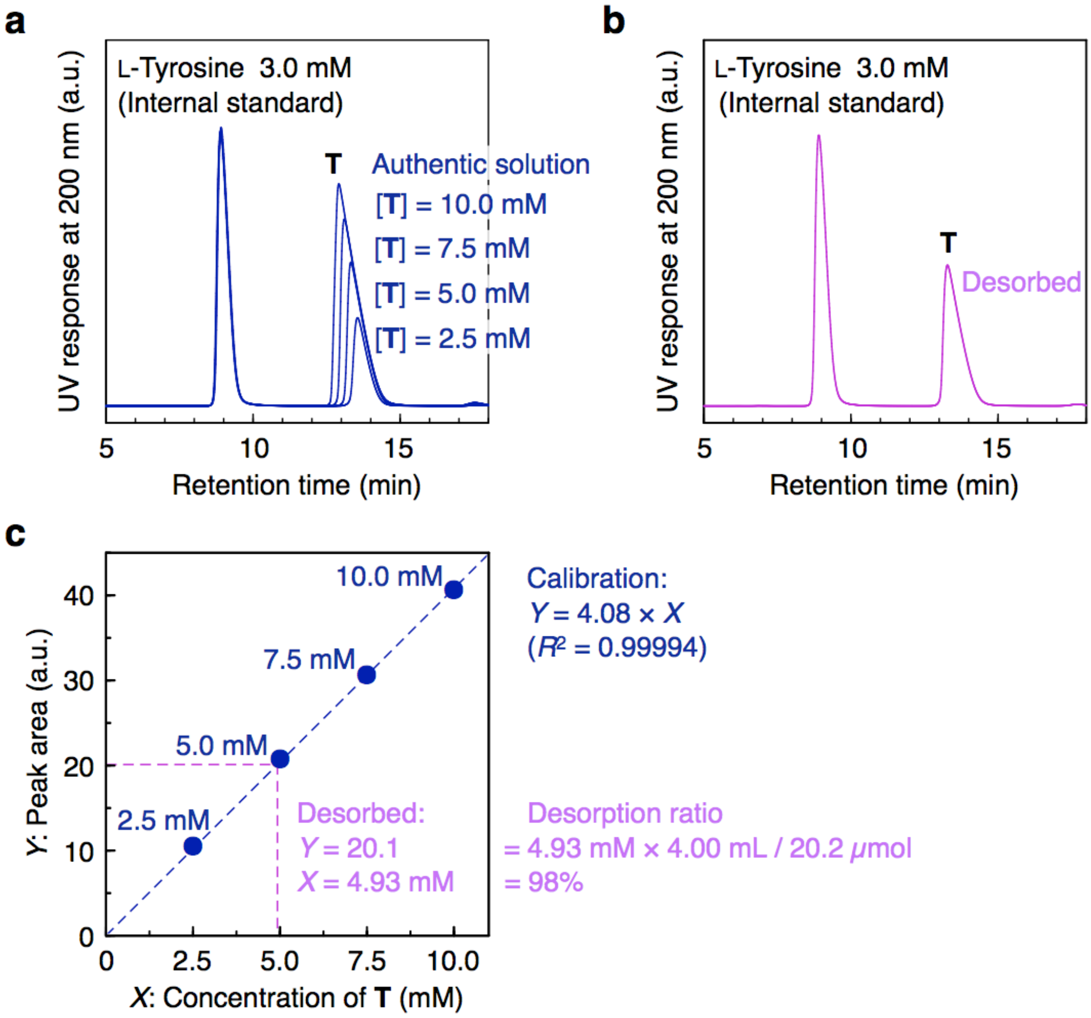

Supplementary Fig. 13 | HPLC quantification of **T** desorbed from the films of poly-**F•T** with a magnetically oriented structure (~10 μm thick, 20.1 mg, containing 20.2 μmol of **T** and the –CO_2H groups). **a**,**b**, HPLC traces of the authentic solutions of **T** (**a**, [**T**] = 2.5, 5.0, 7.5 and 10.0 mM in pH 2.0 aqueous $HClO_4$) and the solution of **T** desorbed from the film of poly-**F•T** (**b**, Methods section in the main text). L-Tyrosine (3.0 mM) was used as an internal standard. **c**, Calibration curve created from the data in **a** (navy) and the desorption ratio of **T** calculated from the data in **b** (magenta).

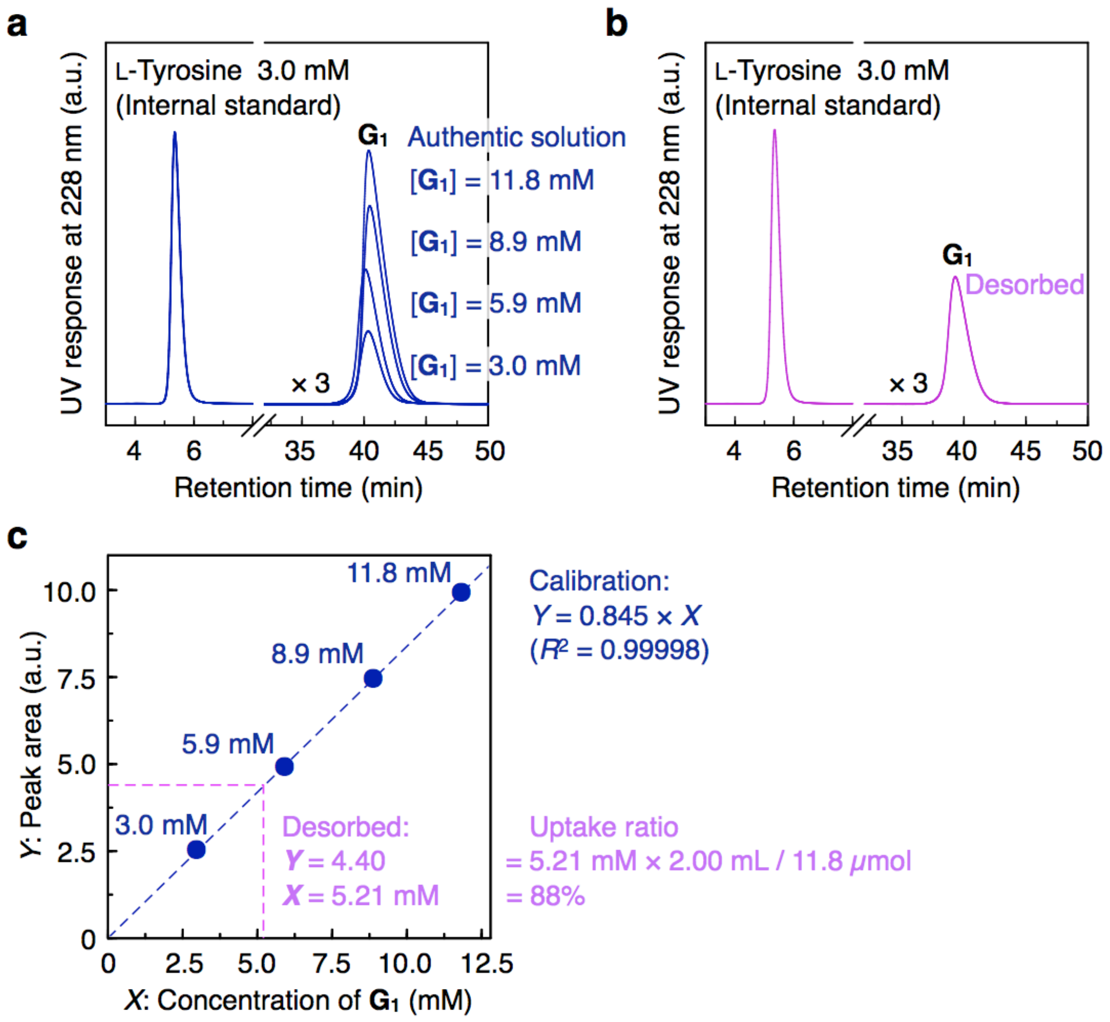

Supplementary Fig. 14 | HPLC quantification of **G**$_1$ adsorbed to the films of poly-**F**•*vacant* with a magnetically oriented structure (~10 μm thick, 10.0 mg, containing 11.8 μmol of the $–CO_2H$ groups). **a**,**b**, HPLC traces of the authentic solutions of **G**$_1$ (**a**, [**G**$_1$] = 3.0, 5.9, 8.9 and 11.8 mM in pH 2.0 aqueous $HClO_4$) and the solution of **G**$_1$ desorbed from the film of poly-**F**•**G**$_1$ (**b**, Methods section in the main text). L-Tyrosine (3.0 mM) was used as an internal standard. **c**, Calibration curve created from the data in **a** (navy) and the uptake ratio of **G**$_1$ calculated from the data in **b** (magenta).

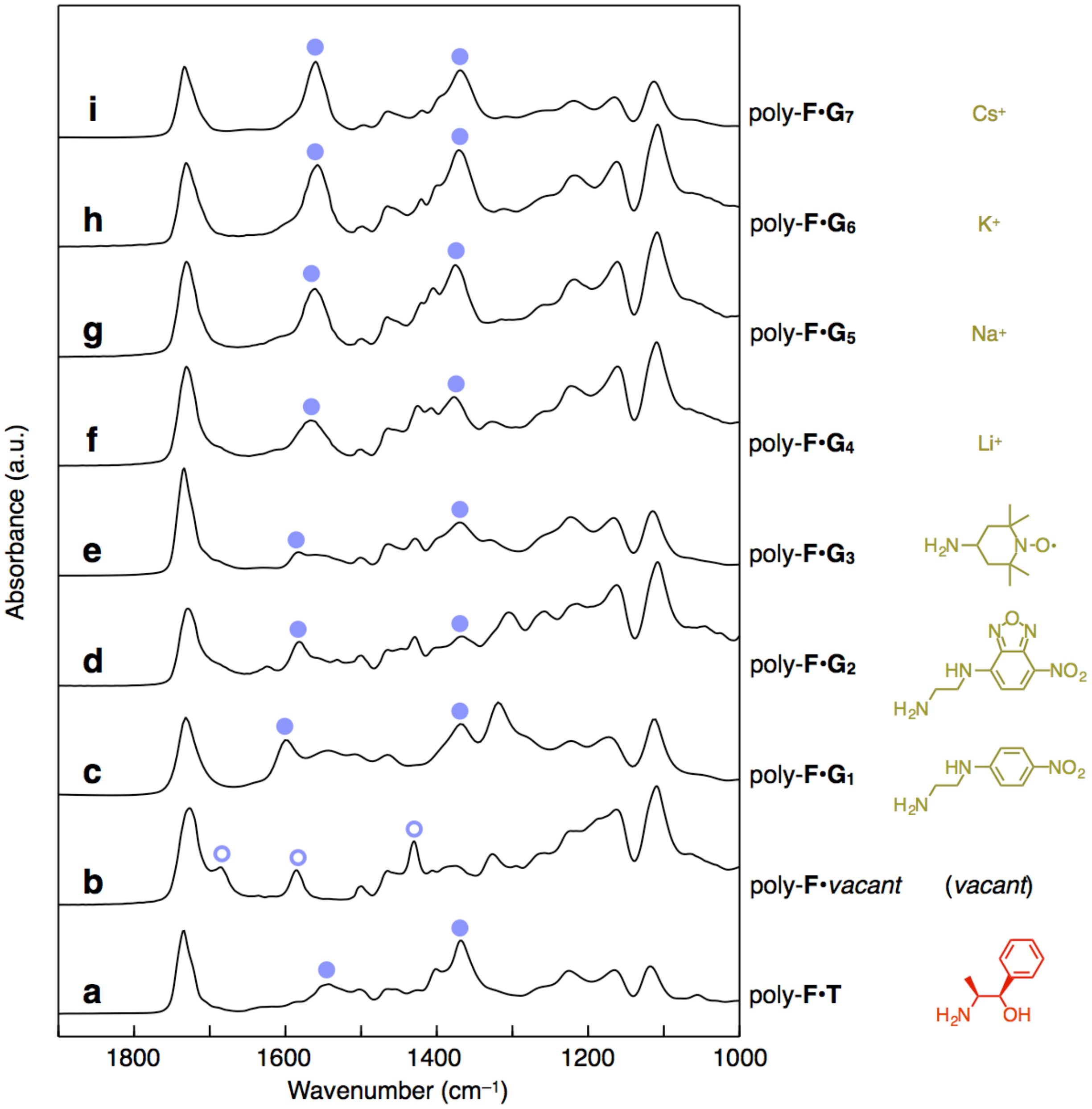

Supplementary Fig. 15 | IR spectra of films of poly-**F** with magnetically oriented structures (~10 μm thick). **a**, poly-**F•T**. **b**, poly-**F•***vacant*. **c**, poly-**F•G$_1$**. **d**, poly-**F•G$_2$**. **e**, poly-**F•G$_3$**. **f**, poly-**F•G$_4$**. **g**, poly-**F•G$_5$**. **h**, poly-**F•G$_6$**. **i**, poly-**F•G$_7$**. Filled circles: absorption attributable to the carboxylate ions forming salt pairs with guest molecules. Open circles: absorption attributable to the free carboxylic acid groups.

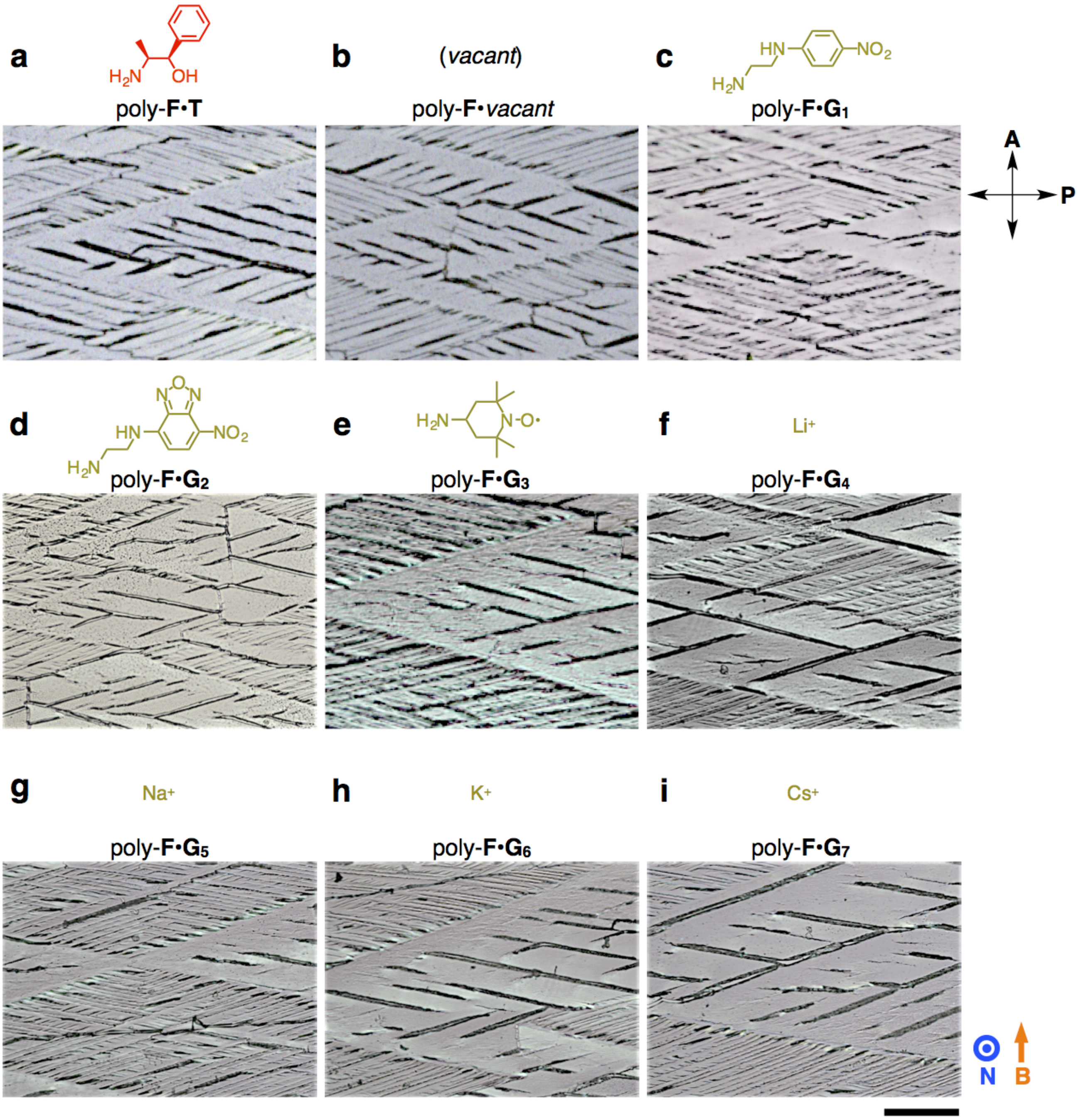

Supplementary Fig. 16 | POM images (under crossed Nicols) of films of poly-**F** with magnetically oriented structures (~10 μm thick). **a**, poly-**F•T**. **b**, poly-**F•***vacant*. **c**, poly-**F•G$_1$**. **d**, poly-**F•G$_2$**. **e**, poly-**F•G$_3$**. **f**, poly-**F•G$_4$**. **g**, poly-**F•G$_5$**. **h**, poly-**F•G$_6$**. **i**, poly-**F•G$_7$**. Scale bar: 200 μm. **B**: Magnetic field applied during the LC film-preparation process. **N**: Normal vector of the film surface. **P**: Polarizer direction. **A**: Analyzer direction.

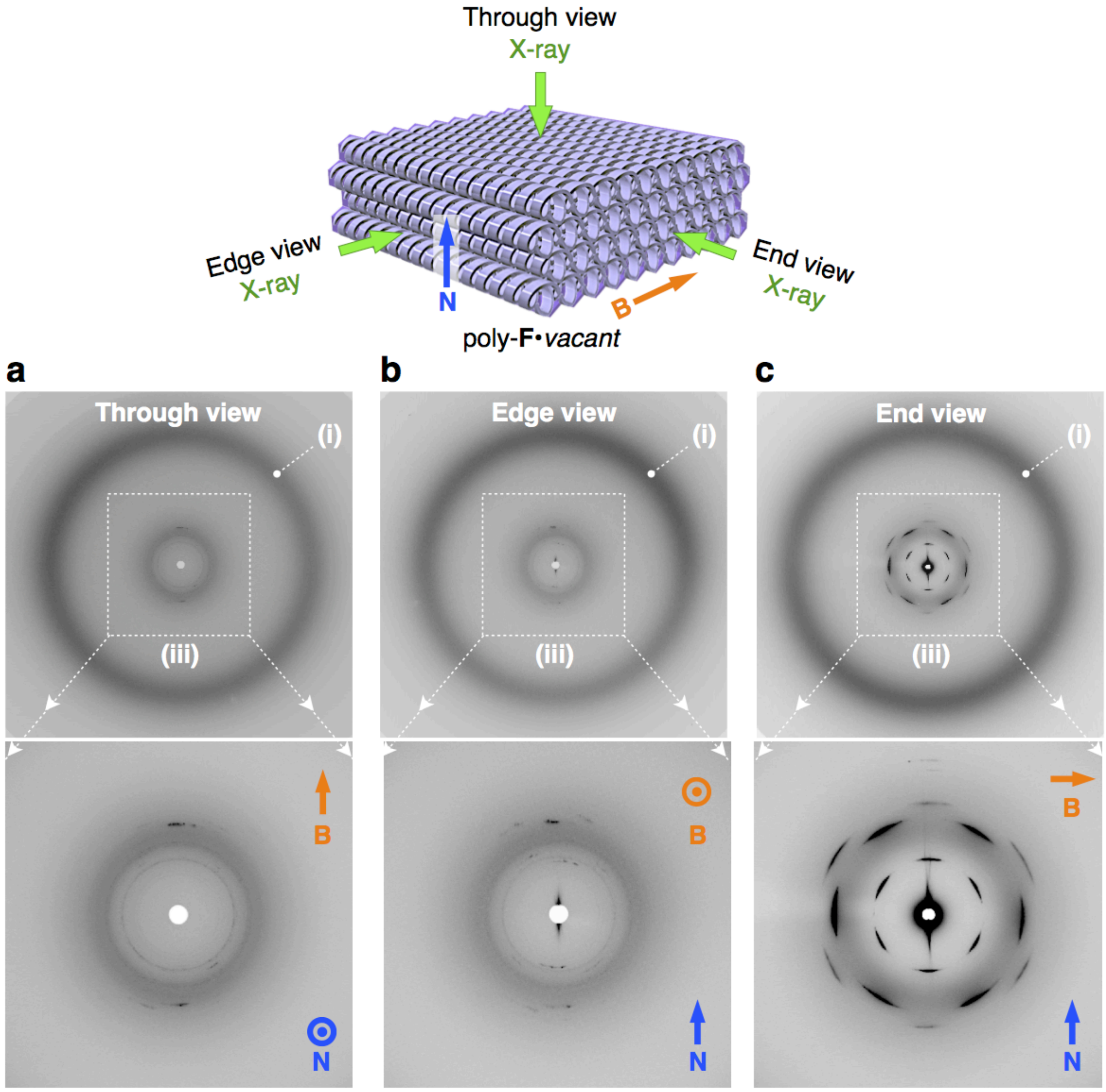

Supplementary Fig. 17 | 2D XRD images of a film of poly-**F**•*vacant* with a magnetically oriented structure (3 mm × 3 mm, ~20 *μ*m thick). **a**, Through view image (X-ray // **N**, X-ray ⊥ **B**). **b**, Edge view image (X-ray ⊥ **N**, X-ray // **B**). **c**, End view image (X-ray ⊥ **N**, X-ray ⊥ **B**). **B**: Magnetic field applied during the LC film-preparation process. **N**: Normal vector of the film surface.

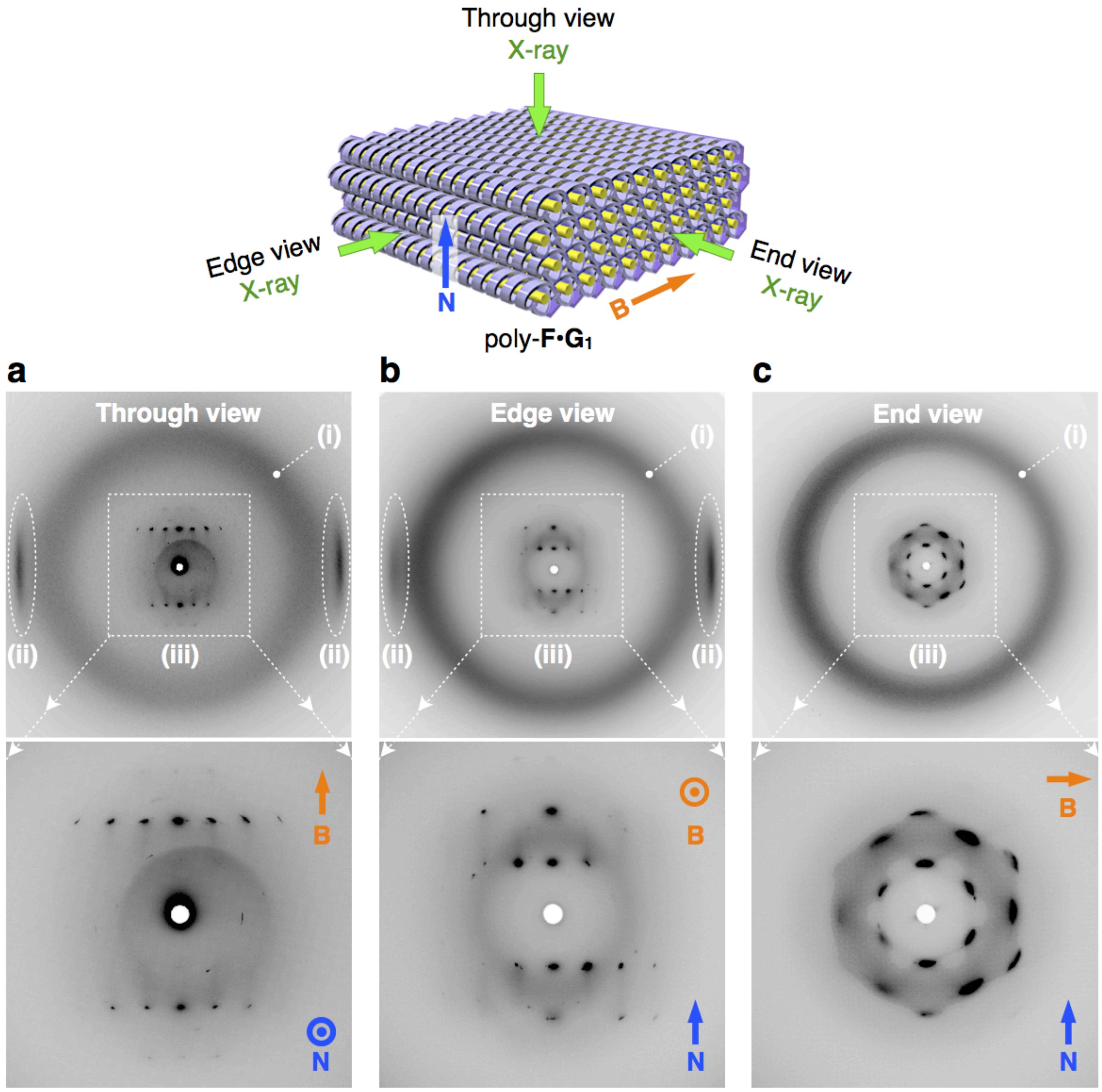

Supplementary Fig. 18 | 2D XRD images of a film of poly-**F**•**G**$_1$ with a magnetically oriented structure (3 mm × 3 mm, ~20 μm thick). **a**, Through view image (X-ray // **N**, X-ray ⊥ **B**). **b**, Edge view image (X-ray ⊥ **N**, X-ray // **B**). **c**, End view image (X-ray ⊥ **N**, X-ray ⊥ **B**). **B**: Magnetic field applied during the LC film-preparation process. **N**: Normal vector of the film surface.

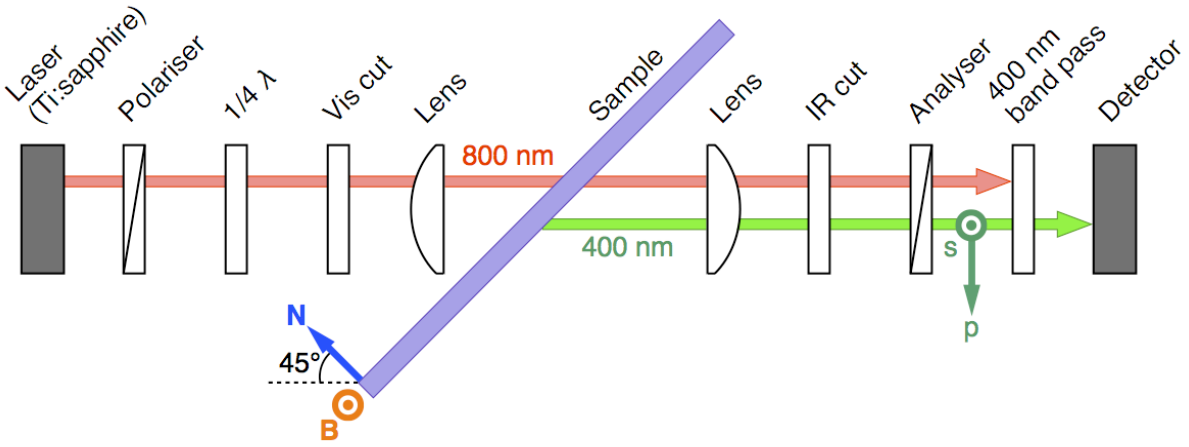

Supplementary Fig. 19 | Optical scheme of the SHG circular dichroism measurement[S3]. As a fundamental beam, circularly polarized light (CPL, 800 nm wavelength) was incident at 45° to a film of poly-**F•G**$_1$. In the case of a film with a magnetically oriented structure (~5 μm thick), the film was set so that the direction of the magnetic field that was applied during the LC film-formation process was perpendicular to the incident plane, *i.e.*, the helical axes of the columnar objects were parallel to the incident plane. Generated frequency-doubled light (400 nm wavelength) was detected from the transmitted direction by using an s- or p-analyzer. **B**: Magnetic field applied during the LC film-preparation process. **N**: Normal vector of the film surface.

Supplementary Table 1 | Thicknesses of film samples.

Measurement	Thickness	Main text	Supplementary Information
POM	~10 μm	Figs 2, 3	Supplementary Figs 1, 3, 4, 16
XRD (polydomain film)	~20 μm	Fig. 4	Supplementary Figs 5, 17, 18
XRD (monodomain clump)	~200 μm	——	Supplementary Figs 8–10
IR	~10 μm	Fig. 3	Supplementary Fig. 15
SHG	~5 μm	Fig. 6	——

Supplementary Table 2 | Elemental analysis for films of poly-F with macroscopically oriented structures.

Sample (preparation)	Composition	Calculated			Found		
		C	H	N	C	H	N
poly-**F•T** (polymerizing **F•T**)	$C_{58} H_{91} N O_{12}$	70.06	9.22	1.41	69.68	9.23	1.30
poly-**F•***vacant* (desorbing **T** from poly-**F•T**)	$C_{49} H_{78} O_{12}$*	69.80*	9.33*	0.00*	69.24	9.51	0.00
poly-**F•G₁** (adsorbing **G₁** to poly-**F•***vacant*)	$C_{56.04} H_{87.68} N_{2.64} O_{12.76}$†	67.13†	8.82†	3.69†	66.22	8.91	3.79
poly-**F•***vacant* (desorbing **G₁** from poly-**F•G₁**)	$C_{49} H_{78} O_{12}$*	69.80*	9.33*	0.00*	68.75	9.47	0.00

* Calculated supposing quantitative desorption of **T** or **G_1**.
† Calculated supposing 88% incorporation of **G_1**.

Supplementary Methods: Determination of the Space Group of poly-F•T

The data collected at BL26B2 in SPring-8 for a monodomain clump of poly-**F•T** were processed with the program CrystalClear (Rigaku)[S4].

Bravais lattice. Within 6 Å resolution of the 18 frames, a total of 183 reflections with $I > 20\sigma(I)$ were found (Supplementary Fig. 5b). The unit cell was determined to be primitive hexagonal or trigonal with a = 31.523(17) Å and c = 42.63(6) Å [V = 36684(4) Å^3], where 163 reflections were indexed with this unit cell.

Laue group. The above unit cell dimension gave the following 7 candidates of the Laue group.

$\bar{1}$, $2/m$, $\bar{3}$, $\bar{3}m1$, $\bar{3}1m$, $6/m$, $6/mmm$

(low <——— symmetry ———> high)

The internal residual factor (R_{int}) was calculated for all candidates, by using the integrated intensity of the indexed 163 reflections[S5]. The most probable Laue group is that with the highest symmetry and with an acceptably small R_{int} value. As a reference, checkCIF (a service of the International Union of Crystallography) does not give an alert when the R_{int} value is less than 0.10[S6]. In the present case, the R_{int} value was 0.026 at $6/mmm$ symmetry, which is the highest symmetry among the candidates. Accordingly, the Laue group was determined to be $6/mmm$.

Space group. The Laue group $6/mmm$ gave the following 18 candidates of space group[S7]:

$P622$, $P6_122$, $P6_222$, $P6_322$, $P6_422$, $P6_522$, $P6mm$, $P6cc$, $P6_3cm$,

$P6_3mc$, $P\bar{6}m2$, $P\bar{6}c2$, $P\bar{6}2m$, $P\bar{6}2c$, $P6/mmm$, $P6/mcc$, $P6_3/mcm$, $P6_3mmc$

From these candidates, the most probable space group was deduced considering the systematic extinction rule for the 0 0 l reflections, as follows[S7], where A and D denote appearance and disappearance of the reflection, respectively.

Reflection condition	0 0 l Reflection						Space group
	0 0 1	0 0 2	0 0 3	0 0 4	0 0 5	0 0 6	
l: any	A	A	A	A	A	A	$P622$, $P6mm$, $P\bar{6}m2$, $P\bar{6}2m$, $P6/mmm$
$l = 2n$	D	A	D	A	D	A	$P6_322$, $P6cc$, $P6_3cm$, P6$_3$mc, $P\bar{6}c2$, $P\bar{6}2c$, $P6_3/mmc$, $P6_3/mcm$, $P6_3/mcc$
$l = 3n$	D	D	A	D	D	A	$P6_222$, $P6_422$
$l = 6n$	D	D	D	D	D	A	$P6_122$, $P6_522$

The 0 0 1, 0 0 2, 0 0 3, 0 0 4, 0 0 5 and 0 0 6 reflections are to be observed at 42.62, 21.32, 14.21, 10.66, 8.52 and 7.11 Å resolution, respectively. Considering that no Bragg spots were detected above 9 Å resolution in all collected frames (Supplementary Fig. 8b), the 0 0 4, 0 0 5 and 0 0 6 reflections were anticipated to be undetectable irrespective of space group; intensity of such high-order reflections are generally low. Meanwhile, the 0 0 1, 0 0 2 and 0 0 3 reflections are not likely to be missed so long as the reflection condition is met, because reflections with higher orders than these (*e.g.*, the 1 1 3 and 0 2 3 reflections) were detected. In all collected frames, the 0 0 1, 0 0 2 and 0 0 3 reflections were not observed (Supplementary Fig. 8b). Among the above candidates of space group, only $P6_122$ and its enantiomorph $P6_522$ can account for the disappearance of the 0 0 1, 0 0 2 and 0 0 3 reflections. Therefore, the space group was determined to be $P6_122$ or $P6_522$.

Supplementary References

S1. PDXL version 2.2.1. Rigaku Corporation, Tokyo, Japan (2014).

S2. Ishida, Y. *et al. J. Am. Chem. Soc.* **132**, 17435–17446 (2010).

S3. Araoka, F. *et al. Phys. Rev. Lett.* **94**, 137801 (2005).

S4. CrystalClear. Rigaku Americas, The Woodlands, Texas, USA and Rigaku Corporation, Tokyo, Japan (2009).

S5. Giacovazzo, C. *Phasing in Crystallography: A Modern Perspective* (Oxford, 2014).

S6. International Union of Crystallography. checkCIF. http://journals.iucr.org/services/cif/checking/RINTA_01.html

S7. Looijenga-Vos, A. & Buerger, M. J. In *Space-Group Symmetry*: *Vol. A of International Tables for Crystallography* (ed. Hahn, T.), Part 3 (Springer, 2005).
